# Therapeutic Application of Monoclonal Antibodies in Pancreatic Cancer: Advances, Challenges and Future Opportunities

**DOI:** 10.3390/cancers13081781

**Published:** 2021-04-08

**Authors:** Gustavo A. Arias-Pinilla, Helmout Modjtahedi

**Affiliations:** 1 Department of Oncology, Sheffield Teaching Hospitals NHS Foundation Trust, Sheffield S10 2JF, UK; gustavo.ariaspinilla@nhs.net; 2 School of Life Sciences, Pharmacy and Chemistry, Kingston University London, Kingston-upon-Thames, Surrey KT1 2EE, UK

**Keywords:** pancreatic cancer, monoclonal antibodies, mAbs, targeted therapy, target antigens

## Abstract

**Simple Summary:**

Pancreatic cancer is a leading cause of cancer death worldwide. In the majority of patients, cancers are diagnosed at advanced stages of disease and are resistant to current treatments. Therefore, more effective and less toxic therapeutic agents are urgently needed. Monoclonal antibody (mAb)-based technology is an important tool in the discovery of novel therapeutic targets and development of novel therapeutic agents including antibody-based drugs. In this article, we review the therapeutic potential of monoclonal antibody-based agents when used as single agents or in combination with other treatments in pancreatic cancer, factors contributing to the poor response to therapy and emerging opportunities for more effective treatment with antibody-based agents.

**Abstract:**

Pancreatic cancer remains as one of the most aggressive cancer types. In the absence of reliable biomarkers for its early detection and more effective therapeutic interventions, pancreatic cancer is projected to become the second leading cause of cancer death in the Western world in the next decade. Therefore, it is essential to discover novel therapeutic targets and to develop more effective and pancreatic cancer-specific therapeutic agents. To date, 45 monoclonal antibodies (mAbs) have been approved for the treatment of patients with a wide range of cancers; however, none has yet been approved for pancreatic cancer. In this comprehensive review, we discuss the FDA approved anticancer mAb-based drugs, the results of preclinical studies and clinical trials with mAbs in pancreatic cancer and the factors contributing to the poor response to antibody therapy (e.g. tumour heterogeneity, desmoplastic stroma). MAb technology is an excellent tool for studying the complex biology of pancreatic cancer, to discover novel therapeutic targets and to develop various forms of antibody-based therapeutic agents and companion diagnostic tests for the selection of patients who are more likely to benefit from such therapy. These should result in the approval and routine use of antibody-based agents for the treatment of pancreatic cancer patients in the future.

## 1. Introduction

Pancreatic ductal adenocarcinoma (PDAC) is one of the most common and aggressive cancer types, with a five-year survival rate of only 2–9% [1]. Worldwide, there were an estimated 458,918 new cases of pancreatic cancer and 432,242 deaths in 2018 [2,3]. In the absence of reliable biomarkers for use in the screening and early detection of pancreatic cancer, and more effective and less toxic therapeutic agents, it has been projected that pancreatic cancer will not only rise in incidence but also will take over breast, prostate and colorectal cancers and become the second leading cause of cancer death in the Western world by 2030 [1,4,5]. 

Treatment of patients with pancreatic cancer involves surgery, chemotherapy and radiotherapy. Although surgery is the only curative treatment, around 80% of patients are diagnosed at late stages of the disease and are not eligible for surgical resection. Adjuvant treatment with chemotherapy is beneficial given the high rate of locoregional relapse after surgery alone. Gemcitabine-based therapy was traditionally the mainstay for treatment of pancreatic cancer [6]. However, the results of the recently published ESPAC-4 trial showed that the combination of gemcitabine plus capecitabine increased median overall survival compared to gemcitabine alone (28.0 vs 25.5 months) with an acceptable toxicity profile, and an estimated 5-year survival of 28.8% for the combination group compared to 16.3% with gemcitabine monotherapy, making this combination the new standard of care in the adjuvant setting [7]. Treatment for borderline resectable or locally advanced unresectable tumours seems to yield better results with chemotherapy (e.g., FOLFIRINOX) rather than chemoradiotherapy, although more robust evidence from trials is needed [8]. 

Patients with metastatic disease are treated with either FOLFIRINOX or gemcitabine plus nab-paclitaxel as first-line in patients with good performance status [9,10]. Erlotinib was approved by the FDA for use in metastatic pancreatic cancer patients based on a study that showed a modest improvement in median survival in patients who received erlotinib plus gemcitabine compared to gemcitabine alone (6.4 vs 5.9 months) but the clinical relevance is controversial [11]. The combination of liposomal irinotecan, fluorouracil and folinic acid (NAPOLI-1 regimen) is the only currently approved second-line chemotherapy for patients with metastatic pancreatic cancer based on a phase 3 trial that showed median overall survival of 6.1 months for the triple combination compared to 4.2 months in patients receiving fluorouracil and folinic acid [12]. Therefore, it is essential to discover novel targets and to develop more effective, less toxic and pancreatic cancer specific therapeutic agents for the long-term benefit of patients with pancreatic cancer.

The advent of hybridoma technology by Köhler and Milstein in 1975, which allows the production of unlimited quantity of an antibody against any target antigen, has revolutionised many areas of biomedical research and medicine [13]. Further technological advances in genetic engineering allowed the production of less immunogenic and more effective types of mAbs (e.g., chimeric, humanised, fully human mAbs, antibody fragments and bispecific antibodies) for use in the treatment of patients with a range of diseases including cancer [14,15,16,17,18]. Indeed, mAb-based therapy is currently one of the two major types of targeted therapy and an attractive therapeutic alternative for the treatment of patients with a wide range of cancers. In this article, we provide a comprehensive review of monoclonal antibody-based agents that have been approved for the treatment of human cancers and the current state of preclinical and clinical studies with monoclonal antibody-based agents in pancreatic cancer. We shall also highlight some of the contributing factors for the poor response to therapy with mAbs, and emerging opportunities for more effective treatment of pancreatic cancer with antibody-based agents in combination with other treatments.

## 2. Therapeutic Antibodies Approved in Cancer 

Over the past few decades, monoclonal antibody-based agents have been approved and used routinely in the treatment of a wide range of human diseases including cancer, infectious, autoimmune and metabolic diseases. Monoclonal antibody-based drugs can be developed by a variety of approaches such as hybridoma technology, phage display technology, the use of transgenic mouse or the single B-cell technique [17,19,20]. 

**Figure 1:**
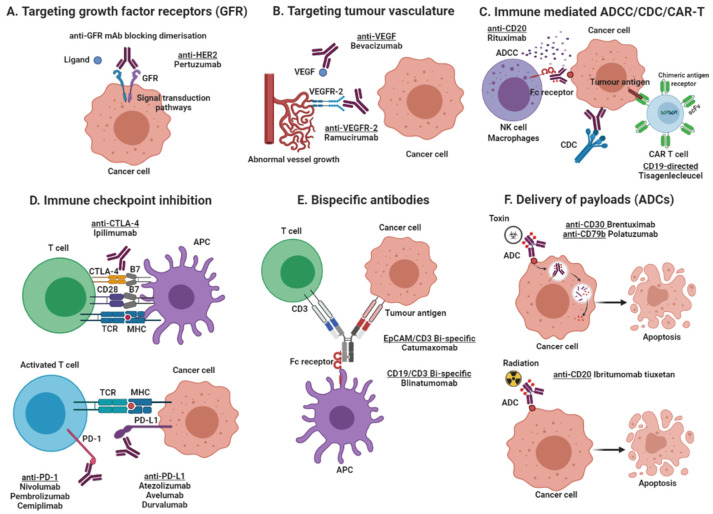
Mechanisms of action of monoclonal antibody-based products. (**A**) Targeting growth factor receptors, blocking the binding of an activating ligand and inhibiting receptor homo- and heterodimerization; (**B**) Targeting of tumour vasculature receptor or its ligands inhibiting angiogenesis; (**C**) Induction of apoptosis by recruitment of immune effector cells (ADCC) or activation of the complement cascade (CDC), and the use of antibody-based molecules to engineer T lymphocytes (CAR T cells); (**D**) Immune checkpoint inhibition by blockade of the PD-1/PD-L1 axis or CTLA-4 inhibitory receptors, increasing cytotoxic T cell activity; (**E**) Simultaneous targeting of two antigens, one on tumour cells and one on effector T cells, by using bispecific antibodies (BITE, bispecific T-cell enhancing); and (**F**) Delivery of payloads such as toxins and radioisotopes to tumour cells. Created with BioRender.com (accessed on 17 March 2021).

Depending on the target antigen and the antibody format, monoclonal antibody-based drugs can produce their anti-tumour activity by several mechanisms (Figure 1). Some mAbs are directed against growth factor receptors with high levels of expression in tumours cells and inhibit tumour growth by blocking the binding of growth factor to its receptor (e.g., anti-EGFR mAbs cetuximab and panitumumab), or by inhibiting receptor dimerization (e.g., anti-HER-2 mAb pertuzumab), consequently inhibiting the downstream cell signalling pathways. In contrast, other antibodies halt tumour growth by inhibiting angiogenesis (e.g., anti-VEGF blocking mAb bevacizumab), stimulating apoptosis (e.g., anti-CD20 mAb rituximab) or delivering lethal doses of radioisotopes (e.g., ibritumomab tiuxetan), or toxins to tumour sites (e.g., brentuximab vedotin, an anti-CD30 mAb conjugated to anti-microtubule agent monomethyl auristatin E). Other mAbs induce tumour killing by immune-mediated antibody-dependent cellular cytotoxicity (ADCC)/complement-dependent cytotoxicity (CDC, e.g., rituximab, trastuzumab) and immune checkpoint inhibition through targeting of PD-1/PD-L1 and CTLA-4 (e.g., nivolumab, pembrolizumab, atezolizumab, ipilimumab). Finally, other therapeutic mAbs are used as component of CAR-T cells, or as bispecific antibodies that induce tumour killing by simultaneous targeting of two different antigens on tumour cells, or bispecific immune cell engager by targeting one antigen on tumour cells and another antigen on T cells (e.g., catumaxomab, blinatumomab, Figure 1).

To date, 45 mAbs have been approved in the USA and/or the European Union (EU) for the treatment of patients with a wide range of cancers (Table 1). In particular, there has been a great deal of research interest in this area in recent years and a growing number of mAb approvals for different indications. Indeed, with the exception of checkpoint inhibitors, nearly half of the approved therapeutic antibodies are directed against one of the following six target antigens: CD19, CD20, the two members of the human epidermal growth factor receptor (HER) family namely EGFR and HER-2, VEGF and VEGFR (Table 1). Interestingly, several immune checkpoint inhibitors such as anti-CTLA-4 mAb ipilumumab, anti-PD-1 mAbs pembrolizumab and nivolumab, and anti-PD-L1 mAbs avelumab and durvalumab have been approved for a wide range of cancer types. Moreover, additional mAbs have been approved outside the USA and EU for treatment of various cancer types including nimotuzumab (in head and neck cancer, nasopharyngeal cancer and glioma) and vivatuxin (in lung cancer) [16]. 

However, despite such advances, to date no antibody-based drugs have been approved for the treatment of patients with pancreatic cancer [21]. Some of the contributing factors are the harsh desmoplastic microenvironment of pancreatic cancer, the heterogeneous nature of tumours and the lack of reliable predictive biomarkers and companion diagnostic tests to select patients who are more likely to respond to such therapy [22,23,24,25]. In the following sections, we discuss the results of preclinical studies and clinical trials with antibody-based agents in pancreatic cancer. We will also highlight the importance of antibody-based technology and other approaches in the discovery of cell surface antigens with high levels of expression in pancreatic cancer (i.e., additional therapeutic targets) and in the development of mAb-based targeted therapy for patients with pancreatic cancer.

## 3. Preclinical Studies 

Over the past few decades, the therapeutic and diagnostic potential of monoclonal antibodies against pancreatic cancer has been investigated both in vitro and in vivo and the results are summarised in Table 2. 

The results from cell proliferation assays in human pancreatic cancer cell lines, cell line-based xenografts and patient-derived tumour xenografts have demonstrated antitumour activity either as single agents or in combination with cytotoxic drugs. The results of in vitro studies have supported the therapeutic potential of mAbs targeting integrin α3 [26], MUC4 [27], MUC1 [28], and MUC13 [29] when used alone or in combination with cytotoxic drugs. Furthermore, mAbs targeting EGFR, TROP2 and α6β4 have been used for radioimmunotherapy and have shown effective localisation of primary tumours and metastatic sites in mouse models [30,31,32].

Moreover, in vivo, naked and conjugated versions of anti-tissue factor mAbs have shown significant antitumour activity in mouse models [33,34,35,36]. Similarly, treatment with mAbs targeting podocalyxin [37], HER2 [38], glypican-1 [39], cell surface plectin 1 [40], galectin-9 [41], RON [42], BAG3 [43], CLDN18.2 [44], mesothelin [45], vimentin [46] and doublecortin-like kinase 1 [47] inhibited the growth of pancreatic tumours in xenograft models. On the other hand, mAbs have also been studied as platforms for cancer theranostics (i.e., a combination of diagnostics and therapeutic) in several animal models [48]. An array of immunoconjugates with radiolabelled antibodies have been used for in vivo imaging using antibody-based PET and SPECT techniques and the results of mAbs targeting CA19.9 and CD147 have shown promise in mouse models [49,50,51]. The main findings are summarized in Table 3. 

## 4. Clinical Trials Evaluating the Diagnostic and Therapeutic Potential of Monoclonal Antibodies in Pancreatic Cancer

**Figure 2:**
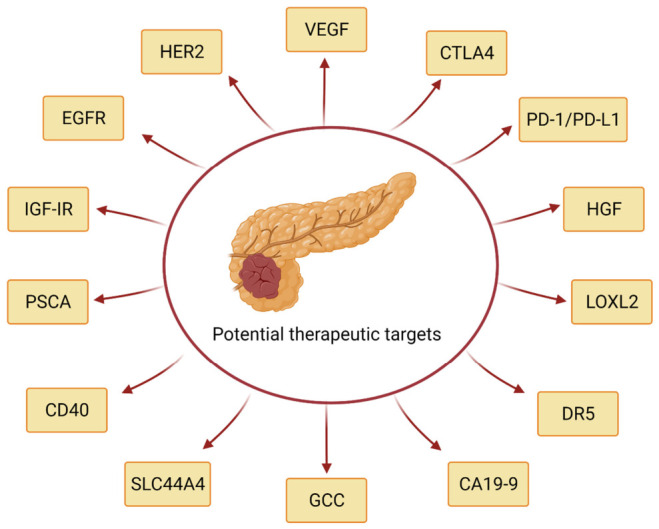
Antigens targeted by antibodies in patients with pancreatic cancer. Created with BioRender.com (accessed on 17 March 2021).

As the results of some preclinical studies summarised in Table 2 were encouraging, several monoclonal antibodies entered different stages of clinical trials in patients with pancreatic cancer. Table 4, Table 5 and Table 6 summarise the results of phase I/II, phase III and ongoing clinical trials with various antibody-based agents (the results of studies in Table 4 and Table 5 are presented in chronological order with the most recent studies at the top of the tables). In the following sections, the antigens targeted by these antibodies (Figure 2), the biological significance of the antigens and the results of completed clinical trials with such antibodies are discussed.

### 4.1. Clinical Trials with Antibodies Targeting Insulin-Like Growth Factor Receptor (IGF-IR) 

Insulin-like growth factor (IGF) signalling activates intracellular pathways such as PI3K (phosphatidyl inositol 3-kinase), Rac, AKT, and MAPK (mitogen-activated protein kinase), regulating the processes involved in cellular proliferation, differentiation and apoptosis. IGF axis has been implicated in various cancer types including pancreatic, breast and prostate cancer, melanoma and Ewing sarcoma and has been associated with the development of resistance to other cancer treatments [68,69,70]. 

Ganitumab (AMG 479) is a fully human IgG1 mAb that binds to the extracellular domain of the type I insulin-like growth factor receptor (IGF-IR), interfering with the binding of IGF-1 and IGF-2 ligands, thus inhibiting downstream signalling including PI3K/Akt pathway and leading to inhibition of tumour cell proliferation and induction of apoptosis [71,72]. In a phase 2 study, ganitumab in combination with gemcitabine showed trend towards improved OS compared to gemcitabine alone in patients with metastatic pancreatic cancer [73], although the GAMMA phase 3 trial failed to further validate those results [74]. 

On the other hand, cixutumumab (IMC-A12), a fully human IgG1/λ mAb against IGF-IR [75], was evaluated in combination with erlotinib and gemcitabine in a phase 1b/2 study in metastatic pancreatic cancer patients but did not demonstrate survival benefit [76]. Furthermore, dalotuzumab (MK-0646), an IgG1 humanised mAb specific to IGF-1R was evaluated in combination with gemcitabine in a phase 1/2 study that demonstrated improved OS but not PFS compared to gemcitabine plus erlotinib [77]. Future trials are needed to evaluate the combination of dalotuzumab with standard of care gemcitabine/nab-paclitaxel and FOLFIRINOX.

### 4.2. Clinical Trials with Anti-Epidermal Growth Factor Receptor (EGFR) Antibodies 

HER family members (which includes EGFR, HER2, HER3 and HER4) are activated by its ligands in an autocrine, paracrine or juxtacrine manner, which involves conformational changes leading to homo- or heterodimerisation of HER members and activation of downstream signalling pathways [70,78]. HER family plays a major role in cancer progression and development, regulating several hallmarks of cancer and therefore becoming an attractive therapeutic target [78]. EGFR (also known as ErbB1/HER1) is commonly upregulated in several cancer types including pancreatic cancer, and thus EGFR-targeted therapies have been developed, including monoclonal antibodies against the extracellular domain of EGFR and tyrosine kinase inhibitors (TKIs) [70,79].

Cetuximab is a recombinant human/mouse chimeric EGFR antagonist mAb of IgG1 isotype that binds to the extracellular ligand-binding domain of the EGFR, blocks the binding of EGF and other ligands to the EGFR and the ligand-induced phosphorylation and activation of EGFR and downstream cell signalling molecules, ultimately leading to tumour growth inhibition and apoptosis [80]. It is FDA approved for the treatment of metastatic colorectal cancer and head and neck squamous cell carcinoma (Table 1). Phase 2 trials in patients with pancreatic cancer showed that the addition of cetuximab to docetaxel/irinotecan or gemcitabine did not offer any survival benefit [81,82]. Similarly, a phase 3 study in patients with advanced pancreatic cancer showed that the addition of cetuximab to gemcitabine did not improve outcomes [83]. In contrast, a phase 2 study reported that cetuximab and bevacizumab in combination with gemcitabine, cisplatin and fluorouracil lengthens overall survival by ~6 months and PFS by ~3 months in patients with advanced pancreatic cancer [84]. However, a recent systematic review and meta-analysis concludes that adding cetuximab to standard therapy for the treatment of pancreatic cancer is not beneficial [85]. Tumour heterogeneity and lack of reliable predictive biomarkers for the response to therapy may be some of the contributing factors for these discouraging results to date. 

Panitumumab is a fully human antibody of IgG2 isotype (i.e. has no ADCC function) to the EGFR. Like cetuximab, it also inhibits the binding of ligands to the EGFR and subsequent downstream cell signalling pathways but it has no ADCC and CDC functions [86]. It is FDA approved for the treatment of patients with metastatic colorectal cancer (Table 1). A phase 1 study showed that the addition of panitumumab to gemcitabine-based chemoradiotherapy has manageable toxicity and suggests some efficacy in patients with locally advanced pancreatic cancer [87]. In addition, a phase 2 trial evaluating the combination of panitumumab, erlotinib and gemcitabine compared with erlotinib and gemcitabine alone in patients with metastatic pancreatic cancer demonstrated significant improvement in overall survival (8.3 vs. 4.2 months) although this regimen was associated with increased toxicity [88]. On the other hand, panitumumab-IRDye800, a fluorescently labelled antibody, which previously was found to be highly sensitive and specific for detection of tumours within 5mm in head and neck cancers [89], demonstrated in a phase 1 trial that is safe and might allow direct visualisation during pancreatic cancer surgery [90].

Nimotuzumab is an IgG1 humanised mAb directed against the extracellular domain of EGFR that has also been reported to induce an adaptive immune response [91]. Phase 2 studies have shown that nimotuzumab is safe and well tolerated alone and in combination with gemcitabine, and the combination regimen showed significant improvement in 1-year OS and PFS rates [92,93]. On the other hand, matuzumab (formerly EMD 72000) is a humanised IgG1 mAb to EGFR that demonstrated being safe and well tolerated in a phase 1 trial in combination with gemcitabine [94]. The results of future clinical trials using more reliable biomarkers for the response to therapy with anti-EGFR mAbs may lead to the approval of anti-EGFR mAbs in a more specific population of pancreatic cancer patients.

### 4.3. Clinical Trials with the Anti-Human Epidermal Growth Factor Receptor 2 (HER2) Antibody Trastuzumab 

HER2, another member of the HER family of growth factor receptor tyrosine kinases, is considered to play a vital role in carcinogenesis. HER2 amplification and/or overexpression has been associated with the development of pancreatic cancer, with reported rates of overexpression ranging between 4 and 50% [70,95].

Trastuzumab is a humanised IgG1 antibody that targets the extracellular domain of HER2, triggers receptor internalisation and degradation, exhibits ADCC activity and inhibits the MAPK and PI3K/Akt pathways leading to increased cell cycle arrest and cell growth suppression [96]. It is FDA approved for treatment of breast cancer and metastatic gastric or gastroesophageal junction adenocarcinoma (Table 1). The results of Phase 1/2 clinical trials of trastuzumab in combination with cetuximab or capecitabine did not demonstrate improvement in objective response and/or survival rates in pancreatic cancer patients [97,98].

### 4.4. Clinical Trials with the Anti-Vascular Endothelial Growth Factor (VEGF) Antibody Bevacizumab 

Vascular endothelial growth factor (VEGF) is a major driver of tumour angiogenesis and as such, has attracted interest for use in cancer therapy. VEGFA is secreted by tumour cells and surrounding stroma and binds to VEGFR2 (VEGF receptor-2) activating the Ras-Raf-MAPK-ERK signalling pathway stimulating the proliferation and survival of endothelial cells, leading to angiogenesis and formation of leaky and structurally abnormal blood vessels [99].

Bevacizumab is a humanised IgG1 monoclonal antibody that targets VEGF, neutralizing the ligand and preventing the interaction with its receptor on the surface of endothelial cells, ultimately inhibiting endothelial proliferation and angiogenesis [86]. It is FDA approved for the treatment of patients with metastatic colorectal cancer, non-small cell lung cancer, glioblastoma, metastatic renal cell carcinoma, metastatic HER2-negative breast cancer, cervical cancer, and epithelial ovarian, fallopian tube, or primary peritoneal cancer (Table 1). Unfortunately, the results of phase 3 trials have shown that the addition of bevacizumab to gemcitabine or to gemcitabine-erlotinib did not improve overall survival in patients with advanced pancreatic cancer [100,101].

### 4.5. Clinical Trials with Anti-Cytotoxic Lymphocyte-Associated Antigen-4 (CTLA-4) 

Cytotoxic lymphocyte-associated antigen-4 (CTLA-4) is an immune checkpoint protein constitutively expressed on regulatory T cells that modulates T cell activation and suppressive properties of APCs (antigen presenting cells). CTLA-4 binds competitively to B7 ligands (CD80 and CD86) expressed on APCs with higher affinity than the co-receptor CD28, which activates naive T cells (Figure 1). CTLA-4 blockade would result in regulatory T cell depletion, T cell enhancement and tumour reduction [102,103].

Ipilimumab is an IgG1 monoclonal antibody against the extracellular domain of CTLA-4 that prevents T cell suppression by inhibitory immune checkpoints, resulting in a potent anti-tumour effect by enhancing effector cells and inhibiting regulatory activity of T cells [86,102]. It has been approved by the FDA for the treatment of patients with melanoma, advanced renal cell carcinoma and microsatellite instability-high (MSI-H) or mismatch repair deficient (dMMR) metastatic colorectal cancer (Table 1). Despite the fact that a phase 2 trial of ipilimumab alone in locally advanced and metastatic pancreatic cancer showed to be ineffective [104], a subsequent study of ipilimumab combined with GM-CSF cell-based vaccines (GVAX) showed improved median overall survival and 1-year OS compared to ipilimumab alone, warranting further studies [105].

On the other hand, tremelimumab (CP-675,206) is a fully human IgG2 mAb directed against CTLA-4 without FDA approvals at present. A phase 1 trial of tremelimumab plus gemcitabine in patients with metastatic pancreatic cancer demonstrated to be safe and tolerable [106], while a phase 2 study with tremelimumab monotherapy did not show activity in metastatic pancreatic cancer patients who had tumour progression following first-line chemotherapy [107].

### 4.6. Clinical Trials with Anti-Programmed Cell Death-1 (PD-1) Receptor and Anti-PD-L1 Ligand Antibodies 

The programmed cell death-1 receptor (PD-1) is expressed on the surface of immune effector cells and upon binding to its ligands, PD-L1 and PD-L2, suppresses proliferation and immune response of T-cells. Activation of this pathway allows evasion of immune response by cancer cells, and blockade of the axis enhances anti-tumour activity [108].

Pembrolizumab is a humanised anti-PD1 IgG4 mAb approved for the treatment of melanoma, non-small cell lung cancer, head and neck squamous cell carcinoma, classical Hodgkin lymphoma, urothelial carcinoma, gastric or gastroesophageal junction adenocarcinoma, cervical cancer, hepatocellular carcinoma, Merkel cell carcinoma, renal cell carcinoma, squamous cell lung cancer, endometrial carcinoma, BCG-unresponsive high-risk non-muscle invasive bladder cancer, tumour mutational burden-high (TMB H) solid tumours, cutaneous squamous cell carcinoma, MSI-H or dMMR colorectal cancer and locally recurrent unresectable or metastatic triple negative breast cancer whose tumours express PD-L1, with a number of indications that is rapidly expanding (Table 1). A phase 1b study of pembrolizumab in combination with the oncolytic virus pelareorep and chemotherapy showed encouraging efficacy [109] and a phase 2 trial is currently ongoing (NCT03723915, Table 6). On the other hand, a phase 1b/2 study of gemcitabine, nab-paclitaxel, and pembrolizumab showed median PFS and OS of 9.1 and 15.0 months respectively, in chemotherapy-naive pancreatic cancer patients, which indicates a slight improvement compared to previous results for gemcitabine and nab-paclitaxel regimens [110]. Pembrolizumab is also currently being evaluated in phase 1 and 2 trials in combination with either olaparib (NCT04548752), paricalcitol +/– gemcitabine and nab-paclitaxel chemotherapy (NCT02930902) or PEGPH20, a PEGylated version of human recombinant PH20 hyaluronidase (NCT03634332, Table 6).

The COMBAT phase 2 trial evaluated the safety and efficacy of pembrolizumab in combination with the CXCR4 antagonist BL-8040 (motixafortide) in patients with metastatic pancreatic cancer and demonstrated disease control rate (DCR) of 34.5%. In the group receiving study drugs as second-line therapy, the median OS was 7.5 months and 6-month survival rate was 56.3%, suggesting that this combination warrants further investigation. Preliminary results show that along with chemotherapy this combination may provide additional benefit (ORR 32%, DCR 77%) [111]. A phase 2 study will be further evaluating the efficacy of pembrolizumab in combination with BL-8040 (NCT02907099).

Nivolumab is a humanised IgG4 anti-PD1 mAb approved for the treatment of melanoma, non-small cell lung cancer, renal cell carcinoma, classical Hodgkin lymphoma, head and neck squamous cell carcinoma, urothelial carcinoma, colorectal cancer, hepatocellular carcinoma, small cell lung cancer, oesophageal squamous cell carcinoma and unresectable malignant pleural mesothelioma (Table 1). A phase 1 study of nivolumab in combination with nab-paclitaxel plus gemcitabine in patients with advanced pancreatic cancer showed that the combination was safe although the efficacy does not support further investigation [112]. Several studies investigating nivolumab in combination with other agents are underway. For instance, a phase 1/2 trial evaluating nivolumab plus mFOLFIRINOX in patients with borderline resectable disease (NCT03970252), a phase 1/2 study investigating the combination of nivolumab plus BMS-813160 (a CCR2/CCR5 dual antagonist) with or without GVAX (an allogeneic GM-CSF–transfected pancreatic tumour vaccine) in locally advanced pancreatic cancer (NCT03767582), and phase 1 trials evaluating the safety and tolerability of the combination of nivolumab with either intratumoural SD-101 (a TLR9 agonist) and radiation therapy (NCT04050085), or SX-682 (a small-molecule dual-inhibitor of chemokine receptors CXCR1 and CXCR2) in patients with metastatic pancreatic cancer (NCT04477343, Table 6).

Clinical trials are also ongoing evaluating anetumab ravtansine (an anti-mesothelin ADC) and nivolumab either alone or in combination with ipilimumab or gemcitabine (NCT03816358), and a phase 1/2 study evaluating the combination of APX005M (a CD40 agonistic mAb), gemcitabine and nab-paclitaxel with or without nivolumab (NCT03214250, Table 6).

On the other hand, durvalumab (MEDI4736) is a human IgG1κ mAb that selectively blocks PD-L1 binding to PD-1 and CD-80, potentiating an immune response to tumour cells [113]. A phase 2 trial evaluating durvalumab with or without tremelimumab in patients with metastatic pancreatic cancer was well tolerated but did not progress further as the threshold for efficacy was not met in the first part of the study [114]. In addition, a phase 1/2 trial evaluating the combination of guadecitabine and durvalumab in patients with advanced hepatocellular carcinoma, pancreatic cancer or cholangiocarcinoma is currently ongoing (NCT03257761, Table 6). 

Other anti-PD1 antibodies are also being evaluated in clinical trials in patients with metastatic pancreatic cancer including a phase 1/2 study of spartalizumab in combination with the anti-IL6 mAb siltuximab (NCT04191421), a phase 2 trial of camrelizumab in combination with nab-paclitaxel and gemcitabine (NCT04498689), a phase 1 study of the mAb SHR-1210 in combination with paclitaxel-albumin and gemcitabine (NCT04181645), a phase 2 trial of dostarlimab (TSR-042) in combination with niraparib (NCT04493060), a phase 1 trial of spartalizumab in combination with nab-paclitaxel, gemcitabine and the anti-IL-1β mAb canakinumab (NCT04581343), a phase 1 study of zimberelimab (AB122) in combination with AB680 (a CD73 inhibitor), nab-paclitaxel and gemcitabine (NCT04104672), and a phase 3 trial of anti-PD-1 antibody plus modified FOLFIRINOX (NCT03983057). See Table 6.

Finally, a phase 2 trial is evaluating INCMGA00012 (a humanised mAb antagonistic to PD-1) in patients with unresectable or metastatic adenosquamous pancreatic cancer, a rare and more aggressive pancreatic cancer subtype, with worse survival outcomes than pancreatic adenocarcinoma (NCT04116073) [115].

### 4.7. Clinical Trial with Anti-Hepatocyte Growth Factor (HGF) Antibody Ficlatuzumab 

Hepatocyte growth factor (HGF) is a glycoprotein produced by mesenchymal cells of stromal origin that, upon binding to its receptor, leads to dimerisation and phosphorylation of c-MET with subsequent activation of signalling pathways such as MAPK and PI3K, regulating cell proliferation, migration and invasion [116]. Expression of HGF and c-MET are upregulated in pancreatic cancer and are associated with poor prognosis [117,118]. 

Ficlatuzumab (AV-299) is a humanised hepatocyte growth factor (HGF) IgG1κ mAb that interferes with the binding of HGF to c-Met receptor tyrosine kinase, inhibiting phosphorylation, cell proliferation, migration and invasion [119]. Ficlatuzumab is currently being evaluated in a phase 1b trial in combination with gemcitabine and nab-paclitaxel in patients with advanced pancreatic cancer (NCT03316599, Table 6).

### 4.8. Clinical Trial with Anti-Lysyl Oxidase-Like 2 (LOXL2) Antibody Simtuzumab 

Lysyl oxidase-like 2 (LOXL2) is an extracellular matrix-remodelling enzyme that catalyses the cross-linking of collagen and elastin components and is expressed in desmoplastic tumours. LOXL2 promotes epithelial-to-mesenchymal transition and downregulates E-cadherin expression in various cancer types [120,121,122]. It is thought to promote tumour angiogenesis and metastases, has been associated with pancreatic cancer progression and has been reported as a prognostic biomarker in patients who have undergone surgical resection [120,121]. Simtuzumab is a humanised IgG4 mAb that targets LOXL2, inhibiting its enzymatic activity. A phase 2 randomised trial evaluating simtuzumab in combination with gemcitabine failed to show improvement in PFS, OS or ORR in patients with metastatic pancreatic cancer [121].

### 4.9. Clinical Trials with Anti-Death Receptor 5 (DR5) Antibodies 

Tumour necrosis factor (TNF)-related apoptosis-inducing ligand (TRAIL) is a transmembrane protein that, upon binding to death receptors DR4 and DR5, activates the caspase cascade leading to apoptotic cell death via the extrinsic pathway [123]. Thus, agonistic antibodies against DR4 and DR5 have shown to induce apoptosis in tumour cells and enhance tumour sensitivity to chemotherapy, radiotherapy and targeted therapy [123].

Conatumumab (AMG655) is a fully human IgG1 antibody to human death receptor 5 (DR5) that induces apoptosis by caspase activation [124]. A phase 2 study of the combination of gemcitabine plus conatumumab in patients with previously untreated metastatic pancreatic adenocarcinoma showed trend towards improved 6-month survival rate (59% compared to 50% in the gemcitabine plus placebo arm) although 12-month survival rate, OS and ORR were not significantly different [73].

Tigatuzumab (CS-1008) is a humanised IgG1 version of TRA-8, a murine agonist mAb to DR5 [125]. A phase 2 study of tigatuzumab in combination with gemcitabine in patients with unresectable or metastatic pancreatic cancer, showed PFS rate at 16 weeks of 52.5% and median OS of 8.2 months. These results are similar to previous studies with gemcitabine in combination with other agents, suggesting that this combination may be clinically active although no definitive conclusions were drawn on the benefit of adding tigatuzumab [126].

### 4.10. Clinical Trials with Anti-CA19-9 Antigen (CA19-9) Antibodies

Carbohydrate antigen 19-9 (CA19-9), also known as sialyl Lewis A (sLe^a^), is the most widely used and best validated diagnostic and prognostic biomarker in pancreatic cancer, being a useful predictor of tumour stage and resectability, response to therapy and overall survival. However, the predictive positive value is low and is therefore not used in the screening of asymptomatic patients. It has been implicated with the pathogenesis of pancreatic cancer, making it an attractive therapeutic target [127,128]. Preliminary phase 1 data of MVT-5873 (HuMab-5B1), a fully human IgG1 mAb targeting sLe^a^, showed encouraging response as single agent or in combination with nab-paclitaxel and gemcitabine in CA19-9 positive pancreatic cancer patients [114]. In addition, the radiolabelled mAb HuMab-5B1 (MVT-2163), which recognises the cancer antigen CA19-9, demonstrated visualisation of primary tumours and metastases by immune-PET in a phase 1 study [129]. Phase 1 and 2 clinical trials evaluating MVT-5873 in patients with CA19-9 overexpressing tumours are currently ongoing (NCT03801915, NCT03118349 and NCT02672917, Table 6). 

### 4.11. Clinical Trial with Anti-Guanylyl Cyclase C (GCC) Antibody 

Guanylyl cyclase C (GCC) is a transmembrane G protein cell surface receptor that plays a role in the regulation of fluid and electrolyte balance. It is highly expressed in colorectal cancer and in around 60–70% of pancreatic cancers [130,131]. TAK-264 (MLN0264) is an antibody-drug conjugate consisting of a fully human IgG1 mAb against GCC, conjugated to MMAE (monomethyl auristatin E) that, once internalised, leads to cell cycle arrest and apoptosis [130]. A phase 2 study of the ADC TAK-264 in patients with advanced or metastatic pancreatic cancer expressing GCC showed a manageable safety profile but low efficacy, which does not support further studies [132]. 

### 4.12. Clinical Trial with Anti-SLC44A4 Antibody ASG-5ME 

SLC44A4 (CTL4) is a protein differentially expressed in prostate and pancreatic cancers with low expression in normal tissues [60]. ASG-5ME is an ADC formed by a human IgG2 antibody against SLC44A4 conjugated with monomethyl auristatin E (MMAE) [60]. A phase 1 study of ASG-5ME in patients with advanced pancreatic and gastric cancers showed that it was well tolerated but had limited efficacy [133].

### 4.13. Clinical Trial with Anti-CD40 Antibody Selicrelumab 

CD40 (cluster of differentiation 40) is a cell surface molecule, member of the tumour necrosis factor family, that is expressed on antigen-presenting cells such as dendritic cells and myeloid cells, and in a variety of cancer types with very low or no expression in normal cells [134]. CP-870,893 (selicrelumab) is a fully human IgG2 CD40-agonist mAb. A phase 1 study of CP-870,893 plus gemcitabine in patients with advanced pancreatic cancer showed that it was well tolerated and was associated with preliminary evidence of efficacy [135].

### 4.14. Clinical Trial with Anti-Prostate Stem Cell Antigen (PSCA) Antibody AGS-1C4D4 

Prostate stem cell antigen (PSCA) is a glycosylphosphatidylinositol (GPI)-anchored cell surface protein associated with various cancer types such as prostate, bladder, gastric and pancreatic cancer, that has been proposed as a biomarker for detection of circulating tumour cells (CTCs) and for cytological examination of specimens in pancreatic cancer patients [136,137]. AGS-1C4D4, a fully human IgG1κ mAb against PSCA, was evaluated in a phase 2 trial in combination with gemcitabine and demonstrated an improvement in 6-month survival rate in the combination arm versus gemcitabine alone (60.9% vs 44.4%) [138].

The list of currently ongoing clinical trials with antibody-based agents in combination with other therapeutics in patients with pancreatic cancer are summarised in Table 6. The results of such trials should help to unravel whether treatment with such agents lead to long-term therapeutic benefits in pancreatic cancer patients and the underlying mechanism of response or resistance to such therapeutic interventions. 

## 5. Challenges and Future Opportunities with Antibody Therapeutics in Pancreatic Cancer

As the results of preclinical and clinical studies discussed above and presented in summary tables suggest, the application of monoclonal antibody-based agents in the treatment of pancreatic cancer is more likely to be successful when used in combination with other therapies such as cytotoxic drugs, other mAbs, cancer vaccines and/or oncolytic viruses. In addition, simultaneous targeting of signalling pathways, the tumour stroma and the incorporation of immune checkpoint inhibitors could yield better results by modifying the immunosuppressive environment of pancreatic tumours [70,177,178,179,180,181]. 

Remarkable responses to immunotherapy have been shown in patients with several types of solid tumours including melanoma [182,183], non-small cell lung cancer [184,185,186] and renal cell carcinoma [184,185,186]. However, immunotherapy with checkpoint inhibitors in unselected pancreatic cancer patients has not demonstrated clinical efficacy, partly due to the strong immunosuppressive tumour microenvironment and the poor antigenicity of tumour-associated antigens that elicit immune response [103,187]. Pancreatic cancer microenvironment is characterised by dense desmoplasia, hypovascularity and scanty immune effector cells [178,188,189]. Therefore, targeting the tumour stroma is of paramount importance to increase drug delivery, promote T-cell infiltration and activation, and overcome the barriers posed by tumour immune-escape mechanisms and the immunosuppressive environment [190]. The CXCRL12/CXCR4 axis has been implicated in all stages of pancreatic cancer development and contributes to survival, metastasis, chemoresistance and the highly atypical pancreatic cancer microenvironment, which makes this disease particularly difficult to treat [191]. Thus, systemic therapies along with simultaneous targeting of the CXCRL12/CXCR4 axis might be an attractive therapeutic approach.

On the other hand, chimeric antigen receptor (CAR) T cell therapy has emerged in recent years as an attractive alternative for cancer treatment (please see Leonardi et al., in this special issue [192]). CD19 targeted CAR T cells have been approved for the treatment of acute lymphoblastic leukaemia [193], relapsed/refractory diffuse large B-cell lymphoma [194,195,196] and relapsed/refractory follicular lymphoma [197]. More recently, the B-cell maturation antigen (BCMA)-directed genetically modified autologous CAR T-cell therapy idecabtagene vicleucel has been approved for relapsed or refractory multiple myeloma [198]. Other studies have evaluated the feasibility of various chimeric antigen receptor (CAR)-modified T cells recognising antigens such as mesothelin [199] and glypican-1 [200] in solid tumours including pancreatic cancer. Currently, several clinical trials are underway with CAR T cells targeting mesothelin (NCT03638193, NCT03323944), CEA (NCT03818165, NCT04037241, NCT02850536) and claudin 18.2 (NCT04404595, NCT04581473) and the results should unravel whether such treatments can be of therapeutic value in patients with pancreatic cancer. In particular, results of clinical trials evaluating the combination of immune checkpoint inhibitors, cancer vaccines and agents that target the immunosuppressive microenvironment in pancreatic cancer are eagerly anticipated. Preclinical studies in subcutaneous and metastatic pancreatic cancer mouse models demonstrated that the combination of a T-cell vaccine, a PD-1 antagonist and a CD40 agonist mAb was able to eradicate most tumours, favouring antitumour immunity by reprogramming immune resistant tumours [201]. This might become a promising approach in the clinical setting.

A better understanding of the biology of pancreatic cancer and the interplay between pancreatic cancer cells, stellate cells and the microenvironment would provide a more solid rationale for the development of new therapeutics including mAb-based agents. Indeed, to improve survival rates, it is clear that there is an urgent need for the discovery of additional cell surface antigens with high levels of expression in patients at different stages of pancreatic cancer. There are currently several approaches for the discovery of membranous proteins such as monoclonal antibody technology or membrane proteomics analysis of isolated proteins by SDS-PAGE and mass spectrometry [66,202]. Indeed, using monoclonal antibody technology and human pancreatic cancer cell lines established from primary tumours and metastatic sites, both as the source of tumour immunogen and in the antibody screening, we have reported recently the development of three novel antibodies. We found that the antigens recognised by these three novel mAbs were CD109, integrin α3 and CD26, with high levels of expression in several human pancreatic cancer cells and Pancreatic Cancer Tissue microarray [26,66]. Indeed, antibody-based screening will help not only in the discovery of additional therapeutic cell surface antigens with high levels of expression at different stages of pancreatic cancer (i.e., therapeutic targets) but also in the development of antibody-based agents for therapy. Moreover, such antibodies would be excellent tools for investigating the diagnostic, prognostic and predictive values of such antigens and investigating their roles in the complex biology of pancreatic cancer [58]. 

## 6. Summary and Concluding Remarks

Pancreatic cancer is one of the deadliest cancer types, with mortality rates that almost equal its incidence. Despite some advances in diagnosis and treatment, its five-year survival rate has not improved substantially over the past few decades. Due to its rising incidence, pancreatic cancer is predicted to become the second leading cause of cancer death by 2030 in many countries [1,4]. Therefore, in order to improve prognosis for patients with pancreatic cancer, it is vital to avoid modifiable risk factors, to discover novel biomarkers/screening methods for its earlier detection, to identify additional targets, and to develop more specific therapeutic agents and companion diagnostic tests for the selection of a more specific population of patients who are more likely to benefit from therapeutic interventions with mAb-based agents. 

The development of monoclonal antibodies against overexpressed cell surface antigens in pancreatic cancer is an attractive strategy for use in both diagnosis and treatment [58]. Since the invention of the hybridoma technology, efforts have been made to develop mAbs for the treatment of different cancer types. Despite success in the application of this form of therapy and subsequent approval by regulatory bodies in haematological malignancies and various solid tumours, none of these mAbs have been approved for pancreatic cancer as of yet (Table 1).

Monoclonal antibodies therapy offers some advantages over other forms of therapy such as target specificity, reduced toxicity and the potential to trigger immune system activation. However, the modest efficacy, the lack of biomarkers predictive of response and the high cost of antibody production are currently some of the major drawbacks of the use of this form of therapy [14,15].

In pancreatic cancer, as well as in other solid tumours, monoclonal antibodies used as single agents have limited efficacy. Therefore, different treatment combinations have been used in an attempt to improve activity and deliver better survival outcomes (Table 4 and Table 5). Various approaches have been postulated including the combination of two or more mAbs directed against different targets, the combination of mAbs with standard of care chemotherapy, the combination of mAbs with radiotherapy to sensitise tumours, the simultaneous targeting of the vasculature and stroma, the targeting of coinhibitory receptors on effector T cells, and the use of bispecific antibodies to bring effector T cells and NK cells in proximity to tumour cells. 

To date, monoclonal antibodies targeting IGF-IR, HER2 and VEGF, or combinations of these, have not shown encouraging results in pancreatic cancer patients [74,76,97,98,100,101]. In contrast, the anti-EGFR mAbs panitumumab and nimotuzumab have shown to improve survival outcomes in combination with chemotherapy and/or the tyrosine kinase inhibitor erlotinib in phase 2 trials [88,92,93]. Finally, while studies with other mAbs targeting LOXL2 and Notch2/3 receptors have not provided support for further studies in pancreatic cancer [121,203], the results of early phase trials evaluating the efficacy of the anti-DR5 mAbs conatumumab and tigatuzumab [73,125], the anti-CD40 mAb selicrelumab [135], and the anti-PSCA mAb AGS-1C4D4 [138] were encouraging and need to be validated in future studies.

Of the antibody-drug conjugates tested in clinical trials in pancreatic cancer patients, none of them showed significant clinical activity, including the anti-SLC44A4 antibody-drug conjugate ASG-5ME [133], the anti-guanylyl cyclase C antibody-drug conjugate TAK-264 [132], the anti-MUC16 antibody-drug conjugate DMUC5754A [204], and the anti-mucin antibody-drug conjugate 90Y-clivatuzumab tetraxetan based on an interim analysis of a phase 3 trial (NCT01956812). Early trials have also shown the potential of mAbs as diagnostic tools in pancreatic cancer. For instance, the use of an anti-CEA antibody as a fluorescent-labelled agent for intraoperative direct visualisation of tumours [205], and the use of the anti-CA19-9 mAb HuMab-5B1 (MVT-2163) as imaging probe, which allows visualisation of primary and metastatic tumours by immuno-PET [129]. Further clinical studies are currently underway (NCT03801915, NCT03118349 and NCT02672917).

Monoclonal antibodies bind with high affinity and specificity to their target antigens and these properties have been exploited for their use as theranostics, whereby tumours are identified by radiolabelled antibodies on imaging and subsequently treated with conjugates targeting the same antigen. See reviews by King et.al. and Dammes & Peer for further detail [206,207]. Antibody-based theranostic pairs have been developed targeting a variety of antigens including carcinoembrionic antigen (CEA) [208], tissue factor [35], CUB domain containing protein 1 (CDCP1) [209] and Met [210]. For instance, Knutson and colleagues have reported the development of a theranostic monoclonal antibody specific for CEA, conjugated to paclitaxel and a PEGylated near-infrared fluorophore (DyLight™ 680-4xPEG, Thermo Fisher Scientific, #46603, Rockford, IL, USA). They demonstrated that this theranostic mAb was able to detect BxPC-3 pancreatic tumour xenografts and inhibit tumour growth in mouse models [208]. On the other hand, Ferreira et.al. reported the use of labelled monoclonal antibody constructs targeting tissue factor that demonstrated high uptake in BxPC-3 tumour xenografts in PET imaging (^86^Y-DTPA-ALT836) and led to slow tumour growth in mice (^90^Y-DTPA-ALT836) [35]. Similarly, the novel human antibody 4A06 (which recognises CDCP1) radiolabelled with zirconium-89 (^89^Zr-4A06) was able to detect CDCP1 expression, while the therapeutic constructs of the antibody (^177^Lu-4A06 and ^225^Ac-4A06) inhibited the growth of pancreatic subcutaneous xenograft tumours in mice [209]. Furthermore, targeting of Met by the mAb onartuzumab labelled with zirconium-89 (^89^Zr) demonstrated tumour uptake of ^89^Zr-DFO-onartuzumab in Met overexpressing subcutaneous and orthotopic pancreatic tumours by immunoPET, while the construct ^177^Lu-DTPA-onartuzumab induced significant tumour growth delay and improved survival in treated animals. ^89^Zr-DFO-onartuzumab was able to predict treatment response to ^177^Lu-DTPA-onartuzumab [210]. Similar theranostic approaches using small molecule inhibitors, peptides or nanoparticles targeting integrin αvβ6 [211], fibroblast activation protein [212,213], GPC1 [214] and IGF1 receptor [215] have also been investigated in mouse models of pancreatic cancer,

Most of the currently ongoing clinical trials for pancreatic cancer treatment are evaluating anti-PD1 antibodies in combination with cytotoxic drugs, other mAbs or antibody-drug conjugates, cancer vaccines or PARP inhibitors (Table 6). While pembrolizumab has shown some evidence of activity in phase 2 trials in combination with chemotherapy [110], or a CXCR4 antagonist plus chemotherapy [111], the combination of nivolumab plus chemotherapy has shown disappointing results in a phase 1 trial [112]. Interestingly, the combination of immune checkpoint inhibitors with GM-CSF cell-based vaccines and the oncolytic virus pelareorep has shown promising results in early phase trials [105,109], and subsequent studies evaluating this approach are underway (NCT03723915 and NCT03767582, Table 6). 

The lack of reliable predictive biomarkers and companion diagnostic tests to identify patients who are more likely to benefit from this form of therapy, and the development of intrinsic or acquired resistance to mAb-based drugs are some of the factors contributing to the poor response to therapy with not only antibody-based agents but also other forms of therapy. In addition, other factors might impact the efficacy of therapeutic antibodies such as impaired tumour penetration and heterogenous distribution in tumours [216]. In an attempt to overcome these, strategies such as smaller antibody fragments (e.g., Fab fragments, single-chain variable fragments and single-domain antibodies, mini-bodies and nanobodies) have been investigated, although they pose unique challenges due to higher clearance rates and significantly shorter half-lives than full-size antibodies [216]. On the other hand, the smaller size antibody fragments are ideal for use in cancer imaging [217,218]. 

In summary, at present, no antibody-based drugs have yet been approved for the treatment of patients with pancreatic cancer. However, we believe that the results of ongoing clinical trials with antibody-based products, the discovery of other cell surface antigens with high levels of expression at different stages of the disease, a better understanding of the complex biology of pancreatic cancer, its microenvironment, the immune system and the mechanisms of resistance, together with technological advances in the development of various forms of antibody-based agents (e.g., bispecific/multi-specific, antibody fragments such as mini-bodies and nanobodies, radiolabelled antibodies, antibody-drug conjugates) would lead to the approval of monoclonal antibody-based products when used alone or in combination with other therapeutic interventions in patients with pancreatic cancer in the near future.

## Figures and Tables

**Table 1:** Monoclonal antibodies approved in the U.S. and/or European Union for cancer treatment.

MAb Generic Name (Trade Name)	Target Antigen/Isotype	Cancer Type Indication	Date of Approval
Rituximab (Rituxan®)	CD20/Chimeric IgG1	B-cell lymphoma, NHL	1997
		Diffuse large B-cell, CD20+, NHL	2006
		CLL	2010
		Previously untreated follicular, CD20+, B-cell NHL	2011
Trastuzumab (Herceptin®)	HER-2/Humanised IgG1	Metastatic breast cancer	1998
		Early-stage breast cancer	2006
		HER2 overexpressing metastatic gastric or GEJ adenoca	2010
Gentuzumab ozogamicin (Mylotarg®)	CD33/Humanised IgG4	AML	2000 *
		Newly diagnosed, relapsed or refractory CD33+ AML	2017
Alemtuzumab (Campath®)	CD52/Humanised IgG1	B-CLL	2001
Ibritumomab tiuxetan (Zevalin®)	CD20/Murine IgG1; conjugated to 90Y	NHL	2002
Tositumomab-I131 (Bexxar®)	CD20/Murine IgG2a; conjugated to 131I	NHL	2003 *
Cetuximab (Erbitux®)	EGFR/Chimeric IgG1	Metastatic CRC	2004
		Locally or regionally advanced HNSCC or recurrent or metastatic HNSCC	2006
		Recurrent locoregional disease and/or metastatic HNSCC (first-line)	2011
		K-ras wild-type, EGFR-expressing metastatic CRC (first-line)	2012
Bevacizumab (Avastin®)	VEGF/Humanised IgG1	Metastatic CRC	2004
		Locally advanced, metastatic or recurrent NSCLC	2006
		Metastatic HER-2 negative breast cancer	2008
		Metastatic RCC	2009
		GBM	2009
		Metastatic CRC	2013
		Persistent, recurrent or metastatic cervical cancer	2014
		Platinum-resistant, recurrent epithelial ovarian, fallopian tube, or primary peritoneal cancer	2014
		Stage III or IV epithelial ovarian, fallopian tube, or primary peritoneal cancer after initial surgical resection	2018
Panitumumab (Vectibix®)	EGFR/Human IgG2	Metastatic CRC	2006
Ofatumumab (Arzerra®)	CD20/Human IgG1	CLL	2009
		CLL (previously untreated)	2014
		Recurrent or progressive CLL	2016
Catumaxomab (Removab®)	EpCAM/ CD3/Bi-specific Rat IgG2b/mouse IgG2a	Malignant ascites (in Europe)	2009
Ipilimumab (Yervoy®)	CTLA-4/Human IgG1	Unresectable or metastatic melanoma	2011
		Cutaneous melanoma with pathological involvement of regional lymph nodes	2015
		Intermediate or poor risk, previously untreated advanced RCC (in combination with nivolumab)	2018
		MSI-H or dMMR metastatic CRC (in combination with nivolumab)	2018
Brentuximab vedotin (Adcentris®)	CD30/Chimeric IgG1; conjugated to monomethyl auristatin E	ALCL and HL	2011
		cHL (as consolidation post-auto-HSCT)	2015
		pcALCL or CD30-expressing MF	2017
		Previously untreated stage III or IV cHL	2018
		Previously untreated systemic ALCL or other CD30-expressing peripheral T-cell lymphomas	2018
Pertuzumab (Perjecta®)	HER-2/Humanised IgG1	HER-2 positive metastatic breast cancer	2012
		HER-2 positive, locally advanced, inflammatory, or early-stage breast cancer (in combination with trastuzumab as neoadjuvant therapy)	2013
		HER-2 positive early breast cancer at high risk or recurrence	2017
Denosumab (Xgeva®)	RANKL/Human IgG2	Unresectable giant cell tumour of bone	2013
Ado-trastuzumab emtansine (Kadcyla®)	HER-2/Humanised IgG1; conjugated to DM1	HER-2 positive, metastatic breast cancer	2013
		HER-2 positive breast cancer with residual invasive disease	2019
Obinutuzumab (Gazyva®)	CD20/Humanised IgG1	CLL (previously untreated)	2013
		FL	2016
		Previously untreated stage II bulky, III or IV FL	2017
Ramucirumab (Cyramza®)	VEGFR-2/Recombinant IgG1	Advanced or metastatic, gastric or GEJ adenocarcinoma	2014
		Metastatic NSCLC	2014
		Metastatic CRC	2015
		HCC	2019
		First-line treatment of metastatic NSCLC (in combination with erlotinib)	2020
Pembrolizumab (Keytruda®)	PD-1 receptor/Humanised IgG4	Unresectable or metastatic melanoma and disease progression following ipilimumab	2014
		Unresectable and metastatic melanoma (initial treatment)	2015
		Metastatic NSCLC	2016
		Recurrent or metastatic HNSCC	2016
		Refractory cHL	2017
		Previously untreated metastatic non-squamous NSCLC	2017
		Locally advanced or metastatic urothelial carcinoma	2017
		Unresectable or metastatic MSI-H or dMMR solid tumours	2017
		Recurrent locally advanced or metastatic gastric or GEJ adenocarcinoma	2017
		Recurrent or metastatic cervical cancer	2018
		Refractory PMBCL	2018
		First-line treatment metastatic non-squamous NSCLC	2018
		First-line treatment metastatic squamous NSCLC	2018
		HCC	2018
		Recurrent locally advanced or metastatic Merkel cell carcinoma	2018
		Melanoma with involvement of lymph nodes following complete resection	2019
		First-line treatment stage III or metastatic NSCLC	2019
		First-line treatment advanced RCC	2019
		Metastatic or unresectable recurrent HNSCC	2019
		Metastatic SCLC	2019
		Advanced oesophageal squamous cell cancer	2019
		Advanced endometrial carcinoma that is not MSI-H or dMMR	2019
		BCG-unresponsive, high-risk, non-muscle invasive bladder cancer with carcinoma in situ with or without papillary tumours	2020
		Unresectable or metastatic tumour mutational burden-high (TMB H) solid tumours	2020
		Recurrent or metastatic CSCC not curable by surgery or radiation	2020
		First-line treatment unresectable or metastatic MSI-H or dMMR CRC	2020
		R/R cHL	2020
		Locally recurrent unresectable or metastatic TNBC whose tumours express PD-L1	2020
Blinatumomab (Blincyto®)	CD19/Bispecific CD19-directed CD3 T-cell engager	Philadelphia chromosome-negative R/R B-cell precursor ALL	2014
		R/R B-cell precursor ALL	2017
		B-cell precursor ALL in first or second complete remission with MRD >/= 0.1%	2018
Nivolumab (Opdivo®)	PD-1 receptor/Human IgG4	Unresectable or metastatic melanoma	2014
		BRAF V600 wild-type, unresectable or metastatic melanoma (in combination with ipilimumab)	2015
		Metastatic NSCLC	2015
		Advanced RCC	2015
		cHL	2016
		Recurrent or metastatic HNSCC	2016
		Locally advanced or metastatic urothelial carcinoma	2017
		MSI-H or dMMR metastatic CRC	2017
		HCC	2017
		Melanoma with involvement of lymph nodes or metastatic disease following complete resection	2017
		Intermediate or poor risk, previously untreated advanced RCC (in combination with ipilimumab)	2018
		Metastatic SCLC	2018
		HCC (in combination with ipilimumab)	2018
		First-line treatment metastatic NSCLC whose tumours express PD-L1 (in combination with ipilimumab)	2020
		Unresectable advanced, recurrent or metastatic oesophageal squamous cell carcinoma (ESCC)	2020
		Unresectable malignant pleural mesothelioma (first-line; in combination with ipilimumab)	2020
		First-line treatment advanced renal cell carcinoma (in combination with cabozantinib)	2021
Dinutuximab (Unituxin®)	GD2/Chimeric IgG1	High-risk neuroblastoma	2015
Daratumumab (Darzalex®)	CD38/Human IgG1	MM	2015
		Newly diagnosed MM ineligible for autologous SCT	2019
		Newly diagnosed MM eligible for autologous SCT	2019
Necitumumab (Portrazza®)	EGFR/Human IgG1	Metastatic squamous NSCLC (first-line)	2015
Elotuzumab (Empliciti®)	SLAMF7/Humanised IgG1	MM	2015
Atezolizumab (Tecentriq®)	PD-L1/Humanised IgG1	Locally advanced or metastatic urothelial carcinoma	2016
		Metastatic NSCLC	2016
		First-line treatment metastatic non-squamous NSCLC	2018
		Unresectable locally-advanced or metastatic TNBC	2019
		Extensive-stage SCLC	2019
		Unresectable or metastatic HCC (in combination with bevacizumab)	2020
		BRAF V600 mutation-positive unresectable or metastatic melanoma	2020
Olaratumab (Lartruvo®)	PDGFRα/Human IgG1	Metastatic soft-tissue sarcoma	2016
Avelumab (Bavencio®)	PD-L1/Human IgG1	Metastatic Merkel cell carcinoma	2017
		Locally advanced or metastatic urothelial carcinoma	2017
		Advanced RCC	2019
		Maintenance treatment in locally advanced or metastatic urothelial carcinoma (UC)	2020
Durvalumab (Imfinzi®)	PD-L1/Human IgG1	Locally advanced or metastatic urothelial carcinoma	2017
		Unresectable stage III NSCLC	2018
		Extensive-stage SCLC	2020
Rituximab+hyaluronidase human (Rituxan hycela®)	CD20/Chimeric IgG1	Follicular lymphoma, DLBCL and CLL	2017
Inotuzumab ozogamicin (Besponsa®)	CD22/Humanised IgG4; conjugated with calicheamicin	R/R B-cell precursor ALL	2017
Mogamulizumab (Poteligeo®)	CCR4/Humanised IgG1	R/R mycosis fungoides or Sezary syndrome	2018
Moxetumomab pasudotox-tdfk (Lumoxiti®)	CD-22/Immunotoxin; IgG1 fragment fused to Pseudomonas exotoxin PE38	R/R hairy cell leukaemia	2018
Cemiplimab-rwlc (Libtayo®)	PD-1/Human IgG4	Metastatic CSCC or locally advanced CSCC who are not candidates for curative surgery or curative radiation	2018
		Locally advanced and metastatic basal cell carcinoma	2021
		First-line treatment of advanced NSCLC whose tumors have high PD-L1 expression	2021
Trastuzumab + hyaluronidase oysk (Herceptin Hylecta®)	HER-2/Humanised IgG1	HER-2 overexpressing breast cancer	2019
Polatuzumab vedotin-piiq (Polivy®)	CD79b-directed ADC; conjugated to MMAE	R/R DLBCL	2019
Enfortumab vedotin-ejfv (Padcev®)	Nectin-4-directed ADC; conjugated to MMAE	Locally advanced or metastatic urothelial cancer	2019
Fam-trastuzumab deruxtecan-nxki (Enhertu®)	HER-2 directed ADC; conjugated to topoisomerase inhibitor	Unresectable or metastatic HER2-positive breast cancer	2019
		Locally advanced or metastatic HER2-positive gastric or GEJ adenocarcinoma	2021
Isatuximab-irfc (Sarclisa®)	CD38	MM	2020
Sacituzumab govitecan-hziy (Trodelvy®)	Trop-2 directed ADC; conjugated to SN-38	Metastatic TNBC	2020
Daratumumab and hyaluronidase-fihj (Darzalex faspro®)	CD38/Human IgG1	Newly diagnosed or R/R MM	2020
Tafasitamab-cxix (Monjuvi®)	CD19/Humanised Fc-modified cytolytic	R/R DLBCL	2020
Belantamab mafodotin-blmf (Blenrep®)	BCMA/ADC conjugated to microtubule inhibitor monomethyl auristatin F (MMAF)	R/R MM	2020
Naxitamab (Danyelza®)	GD2/Humanised IgG1	R/R high-risk neuroblastoma in the bone or bone marrow (in combination with GM-CSF)	2020
Margetuximab-cmkb (Margenza®)	HER2/Fc engineered chimeric IgG1	Metastatic HER2-positive breast cancer	2020

ADC: antibody drug conjugate; ALCL: anaplastic large cell lymphoma; ALL: acute lymphoblastic leukaemia; AML: acute myeloid leukaemia; auto-HSCT: autologous hematopoietic stem cell transplantation; BCG: Bacillus Calmette-Guerin; B-CLL: B-cell chronic lymphocytic leukaemia; BCMA: B-cell maturation antigen; cHL: classical Hodgkin lymphoma; CLL: chronic lymphocytic leukaemia; CRC: colorectal cancer; CSCC: cutaneous squamous cell carcinoma; CTLA-4: cytotoxic T lymphocyte antigen-4; DLBCL: diffuse large B-cell lymphoma; dMMR: mismatch repair deficient; EGFR: epidermal growth factor receptor; EpCAM: epithelial cell adhesion molecule; FL: follicular lymphoma; GBM: glioblastoma multiforme; GD2: surface disialoganglioside GD2; GEJ: gastroesophageal junction; GM-CSF: granulocyte-macrophage colony-stimulating factor; HCC: hepatocellular carcinoma; HER-2: human epidermal growth factor receptor-2; HNSCC: head and neck squamous cell carcinoma; MM: multiple myeloma; MMAE: monomethyl auristatin E; MRD: minimal residual disease; MSI-H: microsatellite instability-high; NHL: non-Hodgkin lymphoma; NSCLC: non-small-cell lung cancer; pcALCL: primary cutaneous anaplastic large cell lymphoma; PDGFRα: platelet-derived growth factor receptor alpha; PD-1: programmed death-1 receptor; PD-L1: programmed death ligand-1; PMBCL: primary mediastinal large B-cell lymphoma; RANKL: receptor activator of nuclear factor-kappa B ligand; RCC: renal cell carcinoma; R/R: relapsed or refractory; SCLC: small cell lung cancer; SCT: stem cell transplant; SLAMF7: signalling lymphocytic activation molecule F7; TNBC: triple-negative breast cancer. VEGF: vascular endothelial growth factor; VEGFR-2: vascular endothelial growth factor receptor 2. * withdrawn; Taken from: https://www.fda.gov/drugs/resources-information-approved-drugs/hematologyoncology-cancer-approvals-safety-notifications. Updated as of 12 March 2021.

**Table 2:** Monoclonal antibodies against pancreatic cancer in preclinical studies for use as therapeutic tools.

mAb Name	Target Antigen	Source/Immunogen	Finding	Ref.
Humanised anti-TF mAb conjugated with MMAE	TF	-	Significantly inhibited tumour growth in an orthotopic xenograft model, and extended survival in a murine peritoneal dissemination model.	[34]
h-RabMab1	Alternatively spliced TF	-	Orthotopic pancreatic tumours in mice treated with h-RabMab1 were 60% smaller than tumours in control group.	[33]
Humanised anti-hTM4SF5 mAb	TM4SF5	Established based on the mouse mAb mEC2-C obtained by immunisation with the cyclic peptide hTM4SF5EC2-C	Reduced cell viability and cell motility, and modulated the expression of EMT markers (vimentin and E-cadherin) in pancreatic cancer cells that endogenously express TM4SF5.	[52]
Panitumumab labelled with ^111^In or ^177^Lu -	EGFR	-	Radioimmunotherapy with panitumumab labelled with Auger electron-emitting ^111^In or β particle-emitting ^177^Lu inhibited the growth of subcutaneous PANC-1 human pancreatic cancer xenografts in mice.	[30]
PcMab-60	PODXL	Soluble PODXL	Demonstrated antitumour activity in Mia PaCa-2 xenograft mouse models.	[37]
7E	IL-20	-	Prolongs survival and attenuates PD-L1 expression in a transgenic mouse and an orthotopic pancreatic cancer model. Combination of 7E and an anti-PD-1 mAb increases efficacy in inhibiting tumour growth in the orthotopic model.	[53]
H_2_Mab-19	HER2	Purified recombinant extracellular domain of HER2	Reduces tumour development in MiaPaCa-2 xenograft model.	[38]
^90^Y-DTPA-ALT836	TF	-	Slow tumour growth compared to the control groups and had significantly smaller tumour volumes 1-day post-injection.	[35]
Anti-GPC-1 mAb conjugated with MMAF	Glypican-1 (GPC-1)	-	Induces significant tumour growth inhibition against GPC-1-positive pancreatic cell lines (BxPC-3 and T3M-4) and patient-derived tumour models.	[39]
KU44.22B	Integrin α3	CFPAC-1 pancreatic cancer cells	Inhibits proliferation of Capan-2 pancreatic cancer cells and increases migration of CFPAC-1 and BxPC-3 cancer cells in vitro.	[26]
6E8 and E9	MUC4	MUC4beta domain	Decreased proliferation and migration of MUC4 expressing pancreatic cancer cells.	[27]
TAB004	MUC1	Pancreatic tumour lysate isolated from an adenocarcinoma developed by a mouse that was transgenic for human MUC1	TAB004 + Lip-MSA-IL-2 significantly improved survival and slowed tumour growth compared to controls in a human MUC1 transgenic mouse model of pancreatic cancer.	[54]
TROP2-IR700	TROP2	-	Photoimmunotherapy with the humanised anti-TROP2 mAb conjugated to the photosensitizer IR700 significantly inhibits tumour growth in pancreatic cancer and cholangiocarcinoma xenografts.	[31]
ZB131	Cell surface plectin 1	-	Decreases tumour volume 5-fold in xenografts, and induces complete tumour regression in subcutaneous syngeneic pancreatic cancer mouse models.	[40]
TAB004	MUC1	-	TAB004 treatment had minimal effect as single agent on most of pancreatic cancer cell lines except for Capan-2. However, when combined with gemcitabine, paclitaxel, or 5-FU, it significantly increased anti-tumour efficacy.	[28]
Anti-Gal-9 mAb	Galectin-9	-	Induced significant reduction in tumour size in orthotopic pancreatic cancer mouse models.	[41]
28H1	FAP	-	The dual-labeled mAb (conjugated with either DTPA for imaging studies or with DTPA and the photosensitizer IRDye700DX for therapy studies) targets pancreatic tumours in mice with good signal-to-background ratios and favourable biodistribution, and it efficiently induces cell death.	[55]
RC68	EGFR	-	The RC68-based antibody-drug conjugates induced death of EGFR-positive pancreatic cancer cell lines and inhibited the growth of BxPC-3 xenografts.	[56]
H-Zt/g4-MMAE	RON	-	Inhibited the growth of pancreatic cancer xenografts regardless of chemoresistance or metastatic status.	[42]
BAG3-H2L4	BAG3	BAG3- multiple antigenic peptides	Significantly inhibits the growth of Mia PaCa-2 pancreatic cancer cell xenografts	[43]
Zolbetuximab	CLDN18.2	-	Slow tumour growth, improve survival and attenuate development of metastases in xenograft models. Gemcitabine enhanced zolbetuximab-induced ADCC.	[44]
1849-ICG conjugate	TF	-	A single dose of 1849-ICG conjugate accompanied by NIR light exposure inhibited tumour growth in vivo without noticeable adverse effects.	[36]
^90^Y-labeled 059-053	CD147	Human antibody library	Combined treatment using 90Y-labeled 059-053 with gemcitabine significantly suppressed tumour growth and prolonged survival with tolerable toxicity in a BxPC-3 xenograft mouse model of refractory pancreatic cancer.	[57]
Amatuximab	Mesothelin	-	Supressed the development of peritoneal metastases and exhibited synergistic killing in combination with gemcitabine in a peritoneal metastatic pancreatic cancer model.	[45]
Anti-hMUC1 antibody	MUC1	rhMUC1-EC192 protein	Specifically targets MUC1-C pancreatic cancer cells in vitro and in vivo and suppresses the growth of tumours in Capan-2 xenogratfs mouse models.	[58]
Pritumumab	Vimentin	B lymphocytes isolated from a regional draining lymph node of a patient with cervical carcinoma	Inhibits subcutaneous pancreatic cancer xenografts models. The binding of the antibody to pancreatic cancer cells and tissue induces ADCC.	[46]
7E3	Neuregulin 1 (NRG1)	rhNRG1β1-EDC	Inhibits migration and growth of pancreatic cancer cells co-cultured with CAFs in orthotopic pancreatic tumour xenografts.	[59]
CBT-15G	Doublecortin-like kinase 1 (DCLK1)	KLH-linked peptides targeting the DCLK1 extracellular domain	Significantly inhibited SW1990 pancreatic cancer xenograft growth.	[47]
^90^Y-ITGA6B4	α6β4	Human antibody library constructed using a phage-display system	A single dose of ^90^Y-ITGA6B4 inhibited tumour growth in mice bearing BxPC-3 human pancreatic cancer xenografts overexpressing α6β4.	[32]
ASG-5ME	SLC44A4	B300.19 cells engineered to express SLC44A4	The ADC induced potent antitumour activity in both cell line– and patient-derived xenograft models of pancreatic and prostate cancers. Combination studies of ASG5ME and nab-paclitaxel increased antitumour activity compared to single agents alone.	[60]
Novel mAbs against AGR2 and C4.4A	AGR2 and C4.4A	Unconjugated antigenic peptides	Reduced tumour growth and metastasis and led to regression of xenograft tumours in mice, resulting in increased survival.	[61]
^90^Y-TSP-A01	Transferrin receptor (TfR)	Human antibody libraries	Induced almost complete response in mice bearing Mia PaCa-2 tumours (high TfR expression), but it had limited efficacy on BxPC-3 tumours (low TfR expression).	[62]
BNC101	LRG5	-	BNC101 as single agent partially inhibited tumour growth in AsPC1 and PANC-1 pancreatic cancer models, while when used in combination with gemcitabine, it significantly inhibited tumour growth in both models. In contrast, BNC101 as monotherapy or in combination with chemotherapy had no antitumour activity in the LGR5 negative BxPC3 xenograft tumours.	[63]
TCC56	MUC13	PAN2 cells	The ADC can be efficiently internalised and induces cell death in TCC-PAN2 cells.	[29]
Pa65-2	CHC	MIA PaCa-2 pancreatic cancer cells	Inhibited tumour growth and angiogenesis in NOD/SCID mice bearing MIA PaCa-2-derived pancreatic cancer xenografts.	[64]

ADCC: antibody-dependent cellular cytotoxicity; AGR2: anterior gradient 2; CAFs: cancer-associated fibroblasts; EGFR: Epidermal growth factor receptor; GPC-1: glypican-1; ICG: indocyanine green; LGR5: leucine-rich repeat-containing G-protein coupled receptor 5; MMAE: monomethyl auristatin E; MMAF: monomethyl auristatin F; MUC1: mucin 1; MUC5AC: mucin 5AC; PC: pancreatic cancer; NIR: near-infrared photoimmunotherapeutic; PODXL: podocalyxin; TfR: transferrin; TF: tissue factor; TM4SF5: transmembrane 4 superfamily member 5 protein; TROP2: tumour-associated calcium signal transducer 2.

**Table 3:** Monoclonal antibodies against pancreatic cancer in preclinical studies for use as diagnostic tools.

mAb Name	Target Antigen	Source/Immunogen	Finding	Ref.
^89^Zr-DFO-anti-γH2AX-TAT	CA19.9	Fully human 5B1 mAb, generated from blood lymphocytes from a patient immunised with a sLea – KLH vaccine	PET imaging allows monitoring of tumour radiobiological response in a BxPC-3 pancreatic ductal adenocarcinoma subcutaneous xenograft mouse model.	[51]
6G5j mAb conjugated to LICOR-IRDye800	CEACAM	-	Selectively labels pancreatic cancer in nude mouse models.	[65]
KU44.22B	Integrin α3	CFPAC-1 pancreatic cancer cells	Immunodetects integrin α3 by IHC in tumour cell pellets and pancreatic cancer tissue microarrays.	[26]
KU44.13A	CD26	CFPAC-1 pancreatic cancer cells	Immunodetects CD26 by IHC in tumour cell pellets and pancreatic cancer tissue microarrays.	[26]
KU42.33C	CD109	BxPC-3 pancreatic cancer cells	Immunodetects CD109 by Western blot and IHC in tumour cell pellets and pancreatic cancer tissue microarrays.	[66]
^89^Zr-Df-ALT-836	TF	-	Demonstrated high binding affinity and TF-specificity, and persistent accumulation in BxPC-3 xenografts in mice.	[67]
^89^Zr-059-053	CD147	Human antibody phage-display library	^89^Zr-059-053 highly accumulated in CD147-expressing tumours and clearly visualised subcutaneous and orthotopic xenografts.	[50]

CEACAM: carcinoembryonic antigen-related cell adhesion molecules; IHC: immunohistochemistry; MUC1: mucin 1; PET: positron emission tomography.

**Table 4:** Phase I/II clinical trials of monoclonal antibodies alone or in combination with other drugs in pancreatic cancer (www.clinicaltrials.gov accessed on 17 March 2021).

Condition/Stage of Disease	Therapeutic Intervention	*n*	Ph	Outcomes	Ref.
Metastatic PC	Durvalumab + tremelimumab vs. durvalumab	65	II	ORR: 3.1 vs. 0% Not enrolled in part B because the threshold for efficacy was not met in part A	[114]
Metastatic PC	Olaratumab + nab-paclitaxel + gemcitabine	10	I	Well tolerated and manageable toxicity	[139]
Advanced PC	Gemcitabine + MK-0646 (arm A) vs. gemcitabine + MK-0646 + erlotinib (arm B) vs. gemcitabine + erlotinib (arm C)	81	I/II	PFS: 1.8 vs. 1.8 vs. 1.9 months OS: 10.4 vs. 7.1 vs. 5.7 months (*p* = 0.02)	[77]
CA19-9 positive PC	MVT-5873 + nab-paclitaxel + gemcitabine	38	I	Single agent MVT-5873 appears safe and tolerable at biologically active doses	[140]
Metastatic PC	Gemcitabine + nab-paclitaxel + pembrolizumab	17	Ib/II	mPFS: 9.1 months mOS: 15.0 months (in chemo-naive)	[110]
Resected PC	Cetuximab or bevacizumab + gemcitabine + chemoradiation	127	II	mOS: 17 months (both arms) DFS: 11 months	[141]
Locally advanced or metastatic PC	Gemcitabine + nimotuzumab vs. gemcitabine + placebo	192	IIb	mOS: 8.6 vs. 6.0 months (*p* = 0.03) mPFS: 5.1 vs. 3.6 months (*p* = 0.02)	[92]
Advanced or metastatic PC	TAK-264 (MLN0264)	43	II	ORR: 3%	[132]
Metastatic PC	Gemcitabine + simtuzumab (700 mg) vs. gemcitabine + simtuzumab (200 mg) vs. gemcitabine + placebo	240	II	mPFS: 3.7 (*p* = 0.73) vs. 3.5 (*p* = 0.61) vs. 3.7 months mOS: 7.6 (*p* = 0.28) vs. 5.9 (*p* = 0.69) vs. 5.7 months ORR: 13.9 (*p* = 0.16) vs. 14.5 (*p* = 0.20) vs. 23.5%	[121]
Refractory colon and PC	NEO-102 (ensituximab)	19 (4 PC)	I	Safe and well tolerated	[142]
Unresectable PC	Bevacizumab + erlotinib + capecitabine + RT	17	I	Safe and well tolerated	[143]
PC not amenable to curative treatment	Bevacizumab + cetuximab + leucovorin + gemcitabine + cisplatin + fluorouracil vs. leucovorin + gemcitabine + cisplatin + fluorouracil	59	II	mOS: 13.2 vs. 6.8 months (*p* = 0.03) TTP: 10.7 vs. 3.1 months (*p* = 0.004)	[84]
Advanced pancreatic and gastric cancers	ASG-5ME	50	I	Well tolerated with limited evidence of antitumour activity	[133]
Locally advanced PC	Neoadjuvant gemcitabine plus capecitabine, followed by either: capecitabine or UFT + RT (A) *or* capecitabine or UFT + cetuximab + RT (B).	17	II	mOS: 15.8 vs. 22.0 months (*p* > 0.05) mPFS: 10.4 vs. 12.7 months (*p* > 0.05)	[144]
Metastatic PC	Irinotecan + docetaxel vs irinotecan + docetaxel + cetuximab	87	II	ORR: 4.5 vs. 7% mPFS: 3.9 vs. 4.5 monthsmOS: 6.5 vs. 5.3 months	[82]
Locally advanced PC	Cetuximab + gemcitabine + RT	34	II	mOS: 15.3 months	[145]
Metastatic PC	90Y-clivatuzumab tetraxetan + gemcitabine vs. 90Y-clivatuzumab tetraxetan alone	58	Ib	mOS: 2.7 vs. 2.6 months (7.9 vs. 3.4 months in patients who received multiple cycles; *p* = 0.004)	[146]
Locally advanced PC	Panitumumab + gemcitabine-based CRT	14	I	Manageable toxicity mPFS: 8.9 months mOS: 12.3 months	[87]
Metastatic PC	Trastuzumab + cetuximab (after failure of first-line gemcitabine)	49	I/II	mPFS: 1.8 months mOS: 4.6 months	[97]
Metastatic PC	Gemcitabine + erlotinib + cixutumumab vs. gemcitabine + erlotinib	116	Ib/II	mPFS: 3.6 vs. 3.6 months (*p* = 0.97) mOS: 7.0 vs. 6.7 months (*p* = 0.64)	[76]
Metastatic PC	Tremelimumab + gemcitabine	34	I	Safe and acceptable tolerability profile	[106]
Metastatic PC	Ganitumab + gemcitabine	6	Ib	Tolerable and acceptable safety profile	[147]
Borderline and locally advanced PC	Neoadjuvant bevacizumab + gemcitabine	30	II	No survival benefit in patients undergoing resection	[148]
Locally advanced or metastatic PC	Gemcitabine + capecitabine + bevacizumab + erlotinib	44	II	ORR: 23% mPFS: 8.4 months mOS: 12.6 months	[149]
Unresectable locally advanced or metastatic PC	Gemcitabine + nimotuzumab	18	-	mOS: 9.3 months mPFS: 3.7 months	[150]
Unresectable or metastatic PC	Tigatuzumab (CS-1008) + gemcitabine	62	II	ORR: 13.1% mPFS: 3.9 months mOS: 8.2 months	[126]
Advanced PC	CP-870,893 + gemcitabine	22	I	Safe and well tolerated mPFS: 5.2 months mOS: 8.4 months ORR: 19%	[135]
Locally advanced or metastatic PC	Ipilimumab vs. ipilimumab + GVAX	30	Ib	mOS: 3.6 vs. 5.7 months (*p* = 0.07)	[105]
Potentially resectable PC	Gemcitabine + bevacizumab followed by RT + bevacizumab	59	II	mOS: 16.8 months (19.7 months after resection) mPFS: 6.6 months (12.9 months after resection)	[151]
R0 or R1-resected PC	Gemcitabine + cetuximab	76	II	DFS at 18 months: 27.1% mDFS: 10.0 months mOS: 22.4 months	[81]
Metastatic PC	Gemcitabine vs. gemcitabine + AGS-1C4D4	196	II	6-month SR: 44.4 vs. 60.9% (*P* = 0.03) mOS: 5.5 vs. 7.6 months (*p* = 0.12) mPFS: 3.2 vs. 3.8 months (*p* = 0.27) ORR: 13.1 vs. 21.6%	[138]
Advanced PC	Everolimus + cetuximab + capecitabine	43	I/II	ORR: 6.5% mOS: 5.0 months	[152]
Advanced PC	Bevacizumab + gemcitabine + 5-FU	42	II	PFS at 6 months: 49% mPFS: 5.9 months mOS: 7.4 months	[153]
Metastatic PC	Gemcitabine + ganitumab vs. gemcitabine + conatumumab vs. gemcitabine + placebo	125	II	6-month SR: 57 vs. 59 vs. 50% mOS: 8.7 vs. 7.5 vs. 5.9 months mPFS: 5.1 vs.. 4.0 vs. 2.1 months ORR: 10 vs. 3 vs. 3%	[73]
Localized or locally advanced PC	Neoadjuvant cetuximab + gemcitabine + IMRT	37	II	mOS: 24.3 (resected patients) vs. 10 months (not resected)	[154]
Stage III or IV PC	90Y-clivatuzumab tetraxetan + low-dose gemcitabine	42	-	The combination is feasible DCR: 58% mOS: 7.7 months	[155]
HER2 overexpressing metastatic PC	Trastuzumab + capecitabine	17	II	PFS after 12 weeks: 23.5% mOS: 6.9 months	[98]
Advanced PC	Bevacizumab + cetuximab + gemcitabine vs. bevacizumab + cetuximab	61	II	mPFS: 3.5 vs. 1.9 months mOS: 5.4 vs. 4.2 months	[156]
Locally advanced or metastatic PC	Gemcitabine + oxaliplatin + cetuximab	41	II	ORR: 24% mPFS: 6.9 months mOS: 11.3 months	[157]
Locally advanced or metastatic PC	Nimotuzumab	56	II	mPFS: 6.7 weeks PFS after 1 year: 10.3% mOS: 18.1 weeks	[93]
Metastatic PC	Cetuximab + gemcitabine + oxaliplatin	64	II	mOS: 263 vs.. 162 days (WT vs. KRAS mutation) mPFS: 104 vs. 118 days (WT vs. KRAS mutation)	[158]
Locally advanced PC	Cetuximab + gemcitabine + oxaliplatin followed by cetuximab + capecitabine + RT	69	II	mOS: 19.2 months 1-year OS: 66%	[159]
Advanced PC	90Y-clivatuzumab tetraxetan	21	I	Well tolerated with manageable hematologic toxicity	[160]
Locally advanced or metastatic PC	Bevacizumab + gemcitabine + oxaliplatin	55	II	mPFS: 4.9 months mOS: 11.9 months ORR: 36%	[161]
Localised PC	Gemcitabine + bevacizumab + RT	32	II	mPFS: 9.9 months mOS: 11.8 months	[162]
Locally advanced PC	Gemcitabine + cetuximab + RT	16	I	Safe and well tolerated mOS: 10.5 months	[163]
Gemcitabine-refractory metastatic PC	Bevacizumab alone vs. bevacizumab + docetaxel	32	II	mPFS: 43 vs. 48 days mOS: 165 vs. 125 days The study was stopped due to futility.	[164]
Locally advanced or metastatic PC	Ipilimumab	27	II	No responders but one subject experienced a delayed response after initial progressive disease.	[104]
Gemcitabine-refractory metastatic PC	Bevacizumab + erlotinib	36	II	OS at 6 months: 22%	[165]
Locally advanced or metastatic PC	Gemcitabine + capecitabine + bevacizumab + erlotinib	20	I	mOS: 12.5 months mPFS: 9.0 months ORR: 50%	[166]
Locally advanced, unresectable PC	Bevacizumab + capecitabine + RT	82	II	mOS: 11.9 months 1-year survival: 47% mPFS: 8.6 months; RR: 26%	[167]
Metastatic or locally advanced unresectable PC	Bevacizumab + gemcitabine + capecitabine	50	II	ORR: 22% mPFS: 5.8 months mOS: 9.8 months	[168]
Metastatic PC	Cetuximab + gemcitabine + oxaliplatin	64	II	ORR: 33% mPFS: 3.9 months mOS: 7.1 months	[169]
Inoperable PC (head)	Radiolabelled anti-CEA I131 KAb201 mAb	25	I/II	ORR: 6% mOS: 5.2 months	[170]
Chemotherapy-naive metastatic PC	Gemcitabine + cisplatin + bevacizumab	52	II	mTTP: 6.6 months mOS: 8.2 months 1-year survival: 36%	[171]
Advanced PC	Cetuximab + gemcitabine + cisplatin vs. gemcitabine + cisplatin alone	84	II	ORR: 17.5 vs. 12.2% (*p* = 0.55) mPFS: 3.4 vs. 4.2 months (*p* = 0.85) mOS: 7.5 vs. 7.8 months (*p* = 0.74)	[172]
Untreated stage III or IV PC	Matuzumab + gemcitabine	17	I	Well tolerated mOS: 3.7 months	[94]
Locally Advanced PC	Bevacizumab + capecitabine-based chemoradiotherapy	48	I	Safe and well tolerated, required capecitabine dose reduction ORR: 20% mOS: 14.4 months	[173]
Advanced PC	Bevacizumab + gemcitabine	52	II	ORR: 21% mPFS: 5.4 months mOS: 8.8 months	[174]
Advanced PC	Cetuximab + gemcitabine	41	II	ORR: 12% mPFS: 3.8 months mOS: 7.1 months	[175]
Unresectable, measurable PC	Murine mAb 17-1A	28	II	Acceptable toxicity. Lack of efficacy of treatment	[176]

CEA: carcinoembryonic antigen; DCR: disease control rate; DFS: disease free survival; GVAX: GM-CSF cell-based vaccines; IMRT: intensity-modulated radiotherapy; mOS: median overall survival; mPFS: median progression-free survival; mTTP: median time to progression; mTTF: median time to treatment failure; n: number of patients; ORR: Objective response rate; PKs: pharmacokinetics; PR: partial response; RAIT: radioimmunotherapy; SD: stable disease; SR: survival rate; TTP: time to progression; WT: wild-type.

**Table 5:** Phase III clinical trials of monoclonal antibodies alone or in combination with other drugs for pancreatic cancer treatment (www.clinicaltrials.gov accessed on 17 March 2021).

Condition/Stage of Disease	Therapeutic Intervention	*n*	Ph	Outcomes	Ref.
Metastatic PC (GAMMA trial)	Gemcitabine + placebo vs.. gemcitabine + ganitumab (12 mg/kg) vs. gemcitabine + ganitumab (20 mg/kg)	800	III	mOS: 7.2 vs. 7.0 (*p* = 0.49) vs. 7.1 months (*p* = 0.40)	[74]
Advanced PC (CALGB 80303 trial)	Gemcitabine + bevacizumab vs. gemcitabine + placebo	602	III	mOS: 5.8 vs. 5.9 months (*p* = 0.95) mPFS: 3.8 vs. 2.9 months (*p* = 0.07)ORR: 13 vs. 10%	[101]
Unresectable locally advanced or metastatic PC (SWOG S0205 trial)	Gemcitabine vs. gemcitabine + cetuximab	745	III	mOS: 5.9 vs. 6.3 months (*p* = 0.19) mPFS: 3.0 vs. 3.4 months (*p* < 0.18) mTTF: 1.8 vs. 2.3 months (*p* < 0.006) ORR: 14 vs. 12% (*p* = 0.59)	[83]
Metastatic PC	Gemcitabine + erlotinib + bevacizumab vs. gemcitabine + erlotinib + placebo	607	III	mOS: 7.1 vs. 6.0 months (*p* < 0.21) mPFS: 4.6 vs. 3.6 months (*p* < 0.0002) ORR: 13.5 vs. 8.6% (*p* < 0.06)	[100]

n: number of patients.

**Table 6:** Selected ongoing clinical trials evaluating monoclonal antibodies alone or in combination with other drugs in pancreatic cancer (www.clinicaltrials.gov accessed on 17 March 2021).

Trials Identifier	Therapeutic Intervention	*n* ^1^	Ph	Study Status (Completion Date)
**Anti-PD-1/PD-L1 mAbs**				
NCT02930902	Pembrolizumab (anti-PD-1 mAb) + paricalcitol vs.. pembrolizumab + paricalcitol + chemotherapy	10	I	Active, not recruiting (12/2022)
NCT03723915	Pembrolizumab (anti-PD-1 mAb) + pelareorep	30	II	Active, not recruiting (06/2021)
NCT04548752	Olaparib + pembrolizumab (anti-PD-1 mAb) vs. olaparib	88	II	Not yet recruiting (03/2025)
NCT02907099	BL-8040 + pembrolizumab (anti-PD-1 mAb)	23	II	Active, not recruiting (12/2022)
NCT03634332	PEGPH20 + pembrolizumab (anti-PD-1 mAb)	35	II	Recruiting (01/2021)
NCT04477343	SX-682 (dual-inhibitor CXCR1/CXCR2) + nivolumab (anti-PD-1 mAb)	20	I	Recruiting (10/2022)
NCT03970252	Nivolumab (anti-PD-1 mAb) + mFOLFIRINOX	36	I, II	Recruiting (04/2022)
NCT04050085	SD-101 (TLR9 agonist) + radiation therapy + nivolumab (anti-PD-1 mAb)	6	I	Recruiting (11/2021)
NCT03767582	Nivolumab + CCR2/CCR5 dual antagonist vs. Nivolumab + GVAX + CCR2/CCR5 dual antagonist	30	I, II	Recruiting (03/2022)
NCT03214250	APX005M (CD40 agonistic mAb) + nivolumab (anti-PD-1 mAb) + gemcitabine + nab-paclitaxel vs. APX005M + gemcitabine + nab-paclitaxel.	129	I, II	Active, not recruiting (09/2022)
NCT03373188	Surgery vs. VX15/2503 + surgery vs. VX15/2503 + ipilimumab (anti-CTLA-4 mAb) + surgery vs. VX15/2503 + nivolumab (anti-PD-1 mAb) + surgery	32	I	Recruiting (12/2022)
NCT04191421	Siltuximab (anti-IL-6) + spartalizumab (anti-PD-1 mAb)	42	I, II	Recruiting (12/2022)
NCT04581343	Canakinumab (anti-IL-1β mAb) + spartalizumab (anti-PD-1 mAb) + nab-paclitaxel + gemcitabine	10	I	Recruiting (03/2022)
NCT04116073	INCMGA00012 (anti-PD-1 mAb)	25	II	Recruiting (08/2028)
NCT03983057	mFOLFIRINOX + anti-PD-1 antibody vs. mFOLFIRINOX	830	III	Recruiting (04/2022)
NCT04498689	Camrelizumab (anti-PD-1 mAb) + nab-paclitaxel + gemcitabine	117	II	Recruiting (12/2022)
NCT03989310	Manganese primed anti-PD-1 antibody + nab-paclitaxel + gemcitabine	20	I, II	Recruiting (03/2021)
NCT04104672	AB680 (CD73 inhibitor) + zimberelimab (anti-PD-1 mAb) + nab-paclitaxel + gemcitabine	150	I	Recruiting (01/2024)
NCT04181645	SHR-1210 (anti-PD-1 mAb) + paclitaxel-albumin + gemcitabine	20	I	Recruiting (07/2022)
NCT04493060	Niraparib + TSR-042 (anti-PD-1 mAb)	20	II	Not yet recruiting (12/2023)
NCT03816358	Anetumab ravtansine (anti-mesothelin ADC) + nivolumab (anti-PD-1 mAb) vs. anetumab ravtansine + nivolumab + ipilimumab vs. anetumab ravtansine + nivolumab + gemcitabine	64	I, II	Recruiting (04/2021)
**Anti-CLDN18.2 mAb**				
NCT03816163	Zolbetuximab (anti-CLDN18.2 mAb) + nab-paclitaxel + gemcitabine vs. nab-paclitaxel + gemcitabine	141	II	Recruiting (10/2022)
**Anti-PDGFRα mAb**				
NCT03086369	Olaratumab (anti-PDGFRα mAb) + nab-paclitaxel + gemcitabine vs. placebo + nab-paclitaxel + gemcitabine	186	Ib, II	Active, not recruiting (01/2022)
**Anti-CA19.9 mAb**				
NCT03801915	MVT-5873 (anti-Sialyl Lewis/CA19.9 mAb)	105	II	Recruiting (12/2023)
NCT03118349	MVT-5873 (anti-Sialyl Lewis/CA19.9 mAb) + MVT-1075	7	I	Active, not recruiting (12/2020)
NCT02672917	MVT-5873 (HuMab-5B1)	108	I	Recruiting (12/2020)
**Anti-OX40 mAb**				
NCT04387071	CMP-001 (TLR9 agonist) + INCAGN01949 (anti-OX40 mAb)	42	I, II	Not yet recruiting (07/2023)
**Anti-HGF mAb**				
NCT03316599	Ficlatuzumab (anti-HGF mAb) + gemcitabine + nab-paclitaxel	26	Ib	Active, not recruiting (11/2023)

^1^ Estimated enrolment. mFOLFIRINOX: modified FOLFIRINOX. Taken from: https://www.clinicaltrials.gov/ct2/home (accessed on 17 March 2021). Updated as of 10 January 2021.

## Data Availability

No new data were created or analyzed in this study. Data sharing is not applicable to this article.

## References

[1] McGuigan A., Kelly P., Turkington R.C., Jones C., Coleman H.G., McCain R.S. (2018). Pancreatic cancer: A review of clinical diagnosis, epidemiology, treatment and outcomes. World J. Gastroenterol..

[2] Bray F., Ferlay J., Soerjomataram I., Siegel R.L., Torre L.A., Jemal A. (2018). Global cancer statistics 2018: GLOBOCAN estimates of incidence and mortality worldwide for 36 cancers in 185 countries. CA Cancer J. Clin..

[3] Ferlay J., Colombet M., Soerjomataram I., Dyba T., Randi G., Bettio M., Gavin A., Visser O., Bray F. (2018). Cancer incidence and mortality patterns in Europe: Estimates for 40 countries and 25 major cancers in 2018. Eur. J. Cancer..

[4] Rahib L., Smith B.D., Aizenberg R., Rosenzweig A.B., Fleshman J.M., Matrisian L.M. (2014). Projecting Cancer Incidence and Deaths to 2030: The Unexpected Burden of Thyroid, Liver, and Pancreas Cancers in the United States. Cancer Res..

[5] Rawla P., Sunkara T., Gaduputi V. (2019). Epidemiology of Pancreatic Cancer: Global Trends, Etiology and Risk Factors. World J. Oncol..

[6] Burris H.A., Moore M.J., Andersen J., Green M.R., Rothenberg M.L., Modiano M.R., Cripps M.C., Portenoy R.K., Storniolo A.M., Tarassoff P. (1997). Improvements in survival and clinical benefit with gemcitabine as first-line therapy for patients with advanced pancreas cancer: A randomized trial. J. Clin. Oncol..

[7] Neoptolemos J.P., Palmer D.H., Ghaneh P., Psarelli E.E., Valle J.W., Halloran C.M., Faluyi O., O’Reilly D.A., Cunningham D., Wadsley J. (2017). Comparison of adjuvant gemcitabine and capecitabine with gemcitabine monotherapy in patients with resected pancreatic cancer (ESPAC-4): A multicentre, open-label, randomised, phase 3 trial. Lancet.

[8] Neoptolemos J.P., Kleeff J., Michl P., Costello E., Greenhalf W., Palmer D.H. (2018). Therapeutic developments in pancreatic cancer: Current and future perspectives. Nat. Rev. Gastroenterol. Hepatol..

[9] Conroy T., Desseigne F., Ychou M., Bouché O., Guimbaud R., Bécouarn Y., Adenis A., Raoul J.L., Gourgou-Bourgade S., de la Fouchardière C. (2011). FOLFIRINOX versus Gemcitabine for Metastatic Pancreatic Cancer. N. Engl. J. Med..

[10] Von Hoff D.D., Ervin T., Arena F.P., Chiorean E.G., Infante J., Moore M., Seay T., Tjulandin S.A., Ma W.W., Saleh M.N. (2013). Increased Survival in Pancreatic Cancer with nab-Paclitaxel plus Gemcitabine. N. Engl. J. Med..

[11] Moore M.J., Goldstein D., Hamm J., Figer A., Hecht J.R., Gallinger S., Au H.J., Murawa P., Walde D., Wolff R.A. (2007). Erlotinib plus gemcitabine compared with gemcitabine alone in patients with advanced pancreatic cancer: A phase III trial of the National Cancer Institute of Canada Clinical Trials Group. J. Clin. Oncol..

[12] Wang-Gillam A., Li C.-P., Bodoky G., Dean A., Shan Y.-S., Jameson G., Macarulla T., Lee K.H., Cunningham D., Blanc J.F. (2016). Nanoliposomal irinotecan with fluorouracil and folinic acid in metastatic pancreatic cancer after previous gemcitabine-based therapy (NAPOLI-1): A global, randomised, open-label, phase 3 trial. Lancet.

[13] Köhler G., Milstein C. (1975). Continuous cultures of fused cells secreting antibody of predefined specificity. Nature.

[14] Modjtahedi H., Ali S., Essapen S. (2012). Therapeutic application of monoclonal antibodies in cancer: Advances and challenges. Br. Med. Bull..

[15] Pillay V., Gan H.K., Scott A.M. (2011). Antibodies in oncology. N. Biotechnol..

[16] Scott A.M., Allison J.P., Wolchok J.D. (2012). Monoclonal antibodies in cancer therapy. Cancer Immun..

[17] Lu R.M., Hwang Y.C., Liu I.J., Lee C.C., Tsai H.Z., Li H.J., Wu H.C. (2020). Development of therapeutic antibodies for the treatment of diseases. J. Biomed. Sci..

[18] Huang S., van Duijnhoven S.M.J., Sijts A., van Elsas A. (2020). Bispecific antibodies targeting dual tumor-associated antigens in cancer therapy. J. Cancer. Res. Clin. Oncol..

[19] Buss N.A.P.S., Henderson S.J., McFarlane M., Shenton J.M., de Haan L. (2012). Monoclonal antibody therapeutics: History and future. Curr. Opin. Pharmacol..

[20] Modjtahedi H. (2005). Monoclonal Antibodies as Therapeutic Agents: Advances and Challenges. Iran. J. Immunol..

[21] Kaplon H., Reichert J.M. (2021). Antibodies to watch in 2021. mAbs.

[22] Neesse A., Bauer C.A., Öhlund D., Lauth M., Buchholz M., Michl. P., Tuveson D.A., Gress T.M. (2019). Stromal biology and therapy in pancreatic cancer: Ready for clinical translation?. Gut.

[23] Cros J., Raffenne J., Couvelard A., Poté N. (2018). Tumor Heterogeneity in Pancreatic Adenocarcinoma. Pathobiology.

[24] Duffy M.J., Crown J. (2013). Companion biomarkers: Paving the pathway to personalized treatment for cancer. Clin. Chem..

[25] Agarwal A., Ressler D., Snyder G. (2015). The current and future state of companion diagnostics. Pharmgenom. Pers. Med..

[26] Arias-Pinilla G.A., Dalgleish A.G., Mudan S., Bagwan I., Walker A.J., Modjtahedi H. (2020). Development and application of two novel monoclonal antibodies against overexpressed CD26 and integrin α3 in human pancreatic cancer. Sci. Rep..

[27] Aithal A., Orzechowski C., Junker W.M., Kshirsagar P., Shah A., Gautam S.K., Varshney G.C., Batra S.K., Jain M. (2019). Targeting MUC4 in pancreatic cancer using non-shed cell surface bound antigenic epitopes. Abstracts of Papers Submitted to the Joint 50th Anniversary Meeting of the American Pancreatic Association and Japan Pancreas Society. Pancreas.

[28] Bose M., Mukherjee P. (2019). A novel antibody blocks anti-apoptotic activity of MUC1 in pancreatic cancer cell lines. Cancer Res..

[29] Nishii Y., Yamaguchi M., Kimura Y., Hasegawa T., Aburatani H., Uchida H., Hirata K., Sakuma Y. (2015). A newly developed anti-Mucin 13 monoclonal antibody targets pancreatic ductal adenocarcinoma cells. Int. J. Oncol..

[30] Aghevlian S., Cai Z., Hedley D., Winnik M.A., Reilly R.M. (2020). Radioimmunotherapy of PANC-1 human pancreatic cancer xenografts in NOD/SCID or NRG mice with Panitumumab labeled with Auger electron emitting, (111)In or β-particle emitting, (177)Lu. EJNMMI Radiopharm. Chem..

[31] Nishimura T., Mitsunaga M., Sawada R., Saruta M., Kobayashi H., Matsumoto N., Kanke T., Yanai H., Nakamura K. (2019). Photoimmunotherapy targeting biliary-pancreatic cancer with humanized anti-TROP2 antibody. Cancer Med..

[32] Aung W., Tsuji A.B., Sudo H., Sugyo A., Ukai Y., Kouda K., Kurosawa Y., Furukawa T., Saga T. (2016). Radioimmunotherapy of pancreatic cancer xenografts in nude mice using 90Y-labeled anti-α6β4 integrin antibody. Oncotarget.

[33] Lewis C., Karve A., Matiash K., Stone T., Desai P., Bogdanov V. (2020). Preclinical in vivo characterization of a first-in-class, fully humanized antibody targeting alternatively spliced tissue factor. Res. Pract. Thromb. Haemost..

[34] Tsumura R., Anzai T., Manabe S., Takashima H., Koga Y., Yasunaga M., Matsumura Y. (2021). Antitumor effect of humanized anti-tissue factor antibody-drug conjugate in a model of peritoneal disseminated pancreatic cancer. Oncol. Rep..

[35] Ferreira C.A., Ehlerding E.B., Rosenkrans Z.T., Jiang D., Sun T., Aluicio-Sarduy E., Engle J.W., Ni D., Cai W. (2020). 86/90Y-Labeled Monoclonal Antibody Targeting Tissue Factor for Pancreatic Cancer Theranostics. Mol. Pharm..

[36] Aung W., Tsuji A.B., Sugyo A., Takashima H., Yasunaga M., Matsumura Y., Higashi T. (2018). Near-infrared photoimmunotherapy of pancreatic cancer using an indocyanine green-labeled anti-tissue factor antibody. World J. Gastroenterol..

[37] Kaneko M.K., Ohishi T., Kawada M., Kato Y. (2020). A cancer-specific anti-podocalyxin monoclonal antibody (60-mG2a-f) exerts antitumor effects in mouse xenograft models of pancreatic carcinoma. Biochem. Biophys. Rep..

[38] Kato Y., Ohishi T., Sano M., Asano T., Sayama Y., Takei J., Kawada M., Kaneko M.K. (2020). H(2)Mab-19 Anti-Human Epidermal Growth Factor Receptor 2 Monoclonal Antibody Therapy Exerts Antitumor Activity in Pancreatic Cancer Xenograft Models. Monoclon. Antib. Immunodiagn. Immunother..

[39] Nishigaki T., Takahashi T., Serada S., Fujimoto M., Ohkawara T., Hara H., Sugase T., Otsuru T., Saito Y., Tsujiiet S. (2020). Anti-glypican-1 antibody-drug conjugate is a potential therapy against pancreatic cancer. Br. J. Cancer.

[40] Dimastromatteo J., Poisonnier A., Perez S., Coussens L., Kelly K. (2019). Therapeutic targeting of cell surface plectin induces anti-cancer immune response and pancreatic cancer regression. Cancer Res..

[41] Chen L., Wang W., Koide A., Bolen J., Miller G., Koide S. (2019). First in class immunotherapy targeting Galectin-9 promotes T-cell activation and anti-tumor response against pancreatic cancer and other solid tumors. Cancer Res..

[42] Yao H.-P., Feng L., Suthe S.R., Chen L.-H., Weng T.-H., Hu C.-Y., Jun E.S., Wu Z.G., Wang W.L., Kim S.C. (2019). Therapeutic efficacy, pharmacokinetic profiles, and toxicological activities of humanized antibody-drug conjugate Zt/g4-MMAE targeting RON receptor tyrosine kinase for cancer therapy. J. Immunother. Cancer.

[43] Basile A., De Marco M., Festa M., Falco A., Iorio V., Guerriero L., Eletto D., Rea D., Arra C., Lamolinara A. (2019). Development of an anti-BAG3 humanized antibody for treatment of pancreatic cancer. Mol. Oncol..

[44] Türeci Ӧ., Mitnacht-Kraus R., Wöll S., Yamada T., Sahin U. (2018). Characterization of zolbetuximab in pancreatic cancer models. Oncoimmunology.

[45] Mizukami T., Kamachi H., Fujii Y., Matsuzawa F., Einama T., Kawamata F., Kobayashi N., Hatanaka Y., Taketomi A. (2018). The anti-mesothelin monoclonal antibody amatuximab enhances the anti-tumor effect of gemcitabine against mesothelin-high expressing pancreatic cancer cells in a peritoneal metastasis mouse model. Oncotarget.

[46] Babic I., Nomura N., Glassy E., Nurmemmedov E., Yenugonda V., Glassy M., Kesari S. (2018). Abstract 3828: Pritumumab mAb binds cell surface expressed vimentin on pancreatic cancer cells and inhibits tumor growth. Cancer Res..

[47] Weygant N., Qu D., May R., Chandrakesan P., Ge Y., Ryan C.D., An G., Schlosser M.J., Bannerman-Menson E., Houchen C.W. (2016). Systemic delivery of CBT-15G DCLK1-targeted monoclonal antibody dramatically decreases tumorigenesis in a xenograft model of pancreatic cancer. Cancer Res. (Chic. Ill.).

[48] Moek K.L., Giesen D., Kok I.C., de Groot D.J.A., Jalving M., Fehrmann R.S.N., Lub-de Hooge M.N., Brouwers A.H., de Vries E.G. (2017). Theranostics Using Antibodies and Antibody-Related Therapeutics. J. Nucl. Med..

[49] England C.G., Hernandez R., Eddine S.B., Cai W. (2016). Molecular Imaging of Pancreatic Cancer with Antibodies. Mol. Pharm..

[50] Sugyo A., Tsuji A.B., Sudo H., Nagatsu K., Koizumi M., Ukai Y., Kurosawa G., Zhang M.R., Kurosawa Y., Saga T. (2013). Evaluation of 89Zr-Labeled Human Anti-CD147 Monoclonal Antibody as a Positron Emission Tomography Probe in a Mouse Model of Pancreatic Cancer. PLoS ONE.

[51] Poty S., Mandleywala K., O’Neill E., Knight J.C., Cornelissen B., Lewis J.S. (2020). 89Zr-PET imaging of DNA double-strand breaks for the early monitoring of response following α- and β-particle radioimmunotherapy in a mouse model of pancreatic ductal adenocarcinoma. Theranostics.

[52] Park S., Kim D., Park J.A., Kwon H.J., Lee Y. (2020). Targeting TM4SF5 with anti-TM4SF5 monoclonal antibody suppresses the growth and motility of human pancreatic cancer cells. Oncol. Lett..

[53] Lu S.-W., Pan H.-C., Hsu Y.-H., Chang K.-C., Wu L.-W., Chen W.-Y., Chang M.S. (2020). IL-20 antagonist suppresses PD-L1 expression and prolongs survival in pancreatic cancer models. Nat. Commun..

[54] Dréau D., Moore L.J., Wu M., Roy L.D., Dillion L., Porter T., Puri R., Momin N., Wittrup K.D., Mukherjee P. (2019). Combining the Specific Anti-MUC1 Antibody TAB004 and Lip-MSA-IL-2 Limits Pancreatic Cancer Progression in Immune Competent Murine Models of Pancreatic Ductal Adenocarcinoma. Front. Oncol..

[55] Smeets E., Dorst D., van Lith S., Freimoser-Grundschober A., Klein C., Trajkovic-Arsic M., Gotthardt M., Siveke J., Aarntzen E.H. (2019). A dual-labeled anti-FAP antibody for imaging and targeted photodynamic therapy of cancer associated fibroblasts in a pancreatic cancer mouse model. Nuklearmedizin.

[56] Li Z., Wang M., Yao X., Luo W., Qu Y., Yu D., Li X., Fang J., Huang C. (2019). Development of a Novel EGFR-Targeting Antibody-Drug Conjugate for Pancreatic Cancer Therapy. Target. Oncol..

[57] Sugyo A., Tsuji A., Sudo H., Koizumi M., Ukai Y., Kurosawa G., Saga T., Higashi T. (2018). Efficacy Evaluation of Combination Treatment Using Gemcitabine and Radioimmunotherapy with 90Y-Labeled Fully Human Anti-CD147 Monoclonal Antibody 059-053 in a BxPC-3 Xenograft Mouse Model of Refractory Pancreatic Cancer. Int. J. Mol. Sci..

[58] Wu G., Maharjan S., Kim D., Kim J.N., Park B.K., Koh H., Moon K., Lee Y., Kwon H.J. (2018). A Novel Monoclonal Antibody Targets Mucin1 and Attenuates Growth in Pancreatic Cancer Model. Int. J. Mol. Sci..

[59] Ogier C., Colombo P.-E., Bousquet C., Canterel-Thouennon L., Sicard P., Garambois V., Thomas G., Gaborit N., Jarlier M., Pirot N. (2018). Targeting the NRG1/HER3 pathway in tumor cells and cancer-associated fibroblasts with an anti-neuregulin 1 antibody inhibits tumor growth in pre-clinical models of pancreatic cancer. Cancer Lett..

[60] Mattie M., Raitano A., Morrison K., Morrison K., An Z., Capo L., Verlinsky A., Leavitt M., Ou J., Nadell R. (2016). The Discovery and Preclinical Development of ASG-5ME, an Antibody-Drug Conjugate Targeting SLC44A4-Positive Epithelial Tumors Including Pancreatic and Prostate Cancer. Mol. Cancer Ther..

[61] Arumugam T., Deng D., Bover L., Wang H., Logsdon C.D., Ramachandran V. (2015). New Blocking Antibodies against Novel AGR2-C4.4A Pathway Reduce Growth and Metastasis of Pancreatic Tumors and Increase Survival in Mice. Mol. Cancer Ther..

[62] Sugyo A., Tsuji A.B., Sudo H., Okada M., Koizumi M., Satoh H., Kurosawa G., Kurosawa Y., Saga T. (2015). Evaluation of Efficacy of Radioimmunotherapy with 90Y-Labeled Fully Human Anti-Transferrin Receptor Monoclonal Antibody in Pancreatic Cancer Mouse Models. PLoS ONE.

[63] Shojaei F., Walsh C., Smith K., Menendez C., Lopez P., Norton J., Iglesias J., Hidalgo M., Reyes C., Chu P. (2015). The LGR5 monoclonal antibody BNC101 has anti-tumor and anti-cancer stem cell activity in pancreatic cancer. Cancer Res..

[64] Tung K.H., Lin C.W., Kuo C.C., Li L.T., Kuo Y.H., Wu H.C. (2013). CHC promotes tumor growth and angiogenesis through regulation of HIF-1α and VEGF signaling. Cancer Lett..

[65] Lwin T., Hollandsworth H.M., Bouvet M., Amirfakhri S., Filemoni F., Hoffman R.M., Singer B., Bouvet M. (2020). Fluorescent anti-carcinoembryonic antigen-related cell adhesion molecule (CEACAM) detects pancreatic cancer at sub-millimeter resolution in mouse models. Society of Surgical Oncology SSO 2020 - International Conference on Surgical Cancer Care. Ann. Surg. Oncol..

[66] Arias-Pinilla G.A., Dalgleish A.G., Mudan S., Bagwan I., Walker A.J., Modjtahedi H. (2018). Development of novel monoclonal antibodies against CD109 overexpressed in human pancreatic cancer. Oncotarget.

[67] Hernandez R., England C.G., Yang Y., Valdovinos H.F., Liu B., Wong H.C., Barnhart T.E., Cai W. (2017). ImmunoPET imaging of tissue factor expression in pancreatic cancer with 89Zr-Df-ALT-836. J. Control. Release.

[68] Simpson A., Petnga W., Macaulay V.M., Weyer-Czernilofsky U., Bogenrieder T. (2017). Insulin-Like Growth Factor (IGF) Pathway Targeting in Cancer: Role of the IGF Axis and Opportunities for Future Combination Studies. Target. Oncol..

[69] Subramani R., Lopez-Valdez R., Arumugam A., Nandy S., Boopalan T., Lakshmanaswamy R. (2014). Targeting insulin-like growth factor 1 receptor inhibits pancreatic cancer growth and metastasis. PLoS ONE.

[70] Ioannou N., Seddon A.M., Dalgleish A., Mackintosh D., Modjtahedi H. (2012). Expression pattern and targeting of HER family members and IGF-IR in pancreatic cancer. Front. Biosci..

[71] Tabernero J., Yoshino T., Cohn A.L., Obermannova R., Bodoky G., Garcia-Carbonero R., Ciuleanu T.E., Portnoy D.C., Van Cutsem E., Grothey A. (2015). Ramucirumab versus placebo in combination with second-line FOLFIRI in patients with metastatic colorectal carcinoma that progressed during or after first-line therapy with bevacizumab, oxaliplatin, and a fluoropyrimidine (RAISE): A randomised, double-blind, multicentre, phase 3 study. Lancet Oncol..

[72] Qu X., Wu Z., Dong W., Zhang T., Wang L., Pang Z., Ma W., Du J. (2017). Update of IGF-1 receptor inhibitor (ganitumab, dalotuzumab, cixutumumab, teprotumumab and figitumumab) effects on cancer therapy. Oncotarget.

[73] Kindler H.L., Richards D.A., Garbo L.E., Garon E.B., Stephenson J.J., Rocha-Lima C.M., Safran H., Chan D., Kocs D.M., Galimi F. (2012). A randomized, placebo-controlled phase 2 study of ganitumab (AMG 479) or conatumumab (AMG 655) in combination with gemcitabine in patients with metastatic pancreatic cancer. Ann. Oncol..

[74] Fuchs C.S., Azevedo S., Okusaka T., Van Laethem J.L., Lipton L.R., Riess H., Szczylik C., Moore M.J., Peeters M., Bodoky G. (2015). A phase 3 randomized, double-blind, placebo-controlled trial of ganitumab or placebo in combination with gemcitabine as first-line therapy for metastatic adenocarcinoma of the pancreas: The GAMMA trial. Ann. Oncol..

[75] McKian K.P., Haluska P. (2009). Cixutumumab. Expert Opin. Investig. Drugs.

[76] Philip P.A., Goldman B., Ramanathan R.K., Lenz H.J., Lowy A.M., Whitehead R.P., Wakatsuki T., Iqbal S., Gaur R., Benedetti J.K. (2014). Dual blockade of epidermal growth factor receptor and insulin-like growth factor receptor–1 signaling in metastatic pancreatic cancer: Phase Ib and randomized phase II trial of gemcitabine, erlotinib, and cixutumumab versus gemcitabine plus erlotinib (SWOG S0727). Cancer.

[77] Abdel-Wahab R., Varadhachary G.R., Bhosale P.R., Wang X., Fogelman D.R., Shroff R.T., Overman M.J., Wolff R.A., Javle M. (2018). Randomized, phase I/II study of gemcitabine plus IGF-1R antagonist (MK-0646) versus gemcitabine plus erlotinib with and without MK-0646 for advanced pancreatic adenocarcinoma. J. Hematol. Oncol..

[78] Kumar R., George B., Campbell M.R., Verma N., Paul A.M., Melo-Alvim C., Ribeiro L., Pillai M.R., da Costa L.M., Moasser M.M. (2020). HER family in cancer progression: From discovery to 2020 and beyond. Adv. Cancer. Res..

[79] Wee P., Wang Z. (2017). Epidermal Growth Factor Receptor Cell Proliferation Signaling Pathways. Cancers.

[80] Kirkwood J.M., Butterfield L.H., Tarhini A.A., Zarour H., Kalinski P., Ferrone S. (2012). Immunotherapy of cancer in 2012. CA Cancer J. Clin..

[81] Fensterer H., Schade-Brittinger C., Müller H.H., Tebbe S., Fass J., Lindig U., Settmacher U., Schmidt W.E., Märten A., Ebert M.P. (2013). Multicenter phase II trial to investigate safety and efficacy of gemcitabine combined with cetuximab as adjuvant therapy in pancreatic cancer (ATIP). Ann. Oncol..

[82] Burtness B., Powell M., Catalano P., Berlin J., Liles D., Chapman A., Mitchell E., Benson A.B. (2016). Randomized Phase II Trial of Irinotecan/Docetaxel or Irinotecan/Docetaxel Plus Cetuximab for Metastatic Pancreatic Cancer: An Eastern Cooperative Oncology Group Study. Am. J. Clin. Oncol..

[83] Philip P.A., Benedetti J., Corless C.L., Wong R., O’Reilly E.M., Flynn P.J., Rowland K.M., Atkins J.N., Mirtsching B.C., Rivkin S.E. (2010). Phase III study comparing gemcitabine plus cetuximab versus gemcitabine in patients with advanced pancreatic adenocarcinoma: Southwest Oncology Group-directed intergroup trial S0205. J. Clin. Oncol..

[84] Tai C.-J., Huang M.-T., Wu C.-H., Wang C.-K., Tai C.-J., Chang C.-C., Hsieh C.-I., Chang Y.-J., Wu C.-J., Kuo L.-J. (2016). Combination of Two Targeted Medications (Bevacizumab Plus Cetuximab) Improve the Therapeutic Response of Pancreatic Carcinoma. Medicine (Baltim.).

[85] Forster T., Huettner F.J., Springfeld C., Loehr M., Kalkum E., Hackbusch M., Hackert T., Diener M.K., Probst P. (2020). Cetuximab in Pancreatic Cancer Therapy: A Systematic Review and Meta-Analysis. Oncology.

[86] Adler M.J., Dimitrov D.S. (2012). Therapeutic antibodies against cancer. Hematol. Oncol. Clin. North. Am..

[87] van Zweeden A.A., van der Vliet H.J., Wilmink J.W., Meijerink M.R., Meijer O.W.M., Bruynzeel A.M.E., van Tienhoven G., Giovannetti E., Kazemier G., Jacobs M.A. (2015). Phase I Clinical Trial to Determine the Feasibility and Maximum Tolerated Dose of Panitumumab to Standard Gemcitabine-Based Chemoradiation in Locally Advanced Pancreatic. Cancer Clin. Cancer Res..

[88] Halfdanarson T.R., Foster N.R., Kim G.P., Meyers J.P., Smyrk T.C., McCullough A.E., Ames M.M., Jaffe J.P., Alberts S.R. (2019). A Phase II Randomized Trial of Panitumumab, Erlotinib, and Gemcitabine Versus Erlotinib and Gemcitabine in Patients with Untreated, Metastatic Pancreatic Adenocarcinoma: North Central Cancer Treatment Group Trial N064B (Alliance). Oncologist.

[89] van Keulen S., van den Berg N.S., Nishio N., Birkeland A., Zhou Q., Lu G., Wang H.W., Middendorf L., Forouzanfar T., Martin B.A. (2019). Rapid, non-invasive fluorescence margin assessment: Optical specimen mapping in oral squamous cell carcinoma. Oral Oncol..

[90] Lu G., van den Berg N.S., Martin B.A., Nishio N., Hart Z.P., van Keulen S., Fakurnejad S., Chirita S.U., Raymundo R.C., Yi G. (2020). Tumour-specific fluorescence-guided surgery for pancreatic cancer using panitumumab-IRDye800CW: A phase 1 single-centre, open-label, single-arm, dose-escalation study. Lancet Gastroenterol. Hepatol..

[91] Mazorra Z., Chao L., Lavastida A., Sanchez B., Ramos M., Iznaga N., Crombet T. (2018). Nimotuzumab: Beyond the EGFR signaling cascade inhibition. Semin. Oncol..

[92] Schultheis B., Reuter D., Ebert M.P., Siveke J., Kerkhoff A., Berdel W.E., Hofheinz R., Behringer D.M., Schmidt W.E., Goker E. (2017). Gemcitabine combined with the monoclonal antibody nimotuzumab is an active first-line regimen in KRAS wildtype patients with locally advanced or metastatic pancreatic cancer: A multicenter, randomized phase IIb study. Ann. Oncol..

[93] Strumberg D., Schultheis B., Scheulen M.E., Hilger R.A., Krauss J., Marschner N., Lordick F., Bach F., Reuter D., Edler L. (2012). Phase II study of nimotuzumab, a humanized monoclonal anti-epidermal growth factor receptor (EGFR) antibody, in patients with locally advanced or metastatic pancreatic cancer. Invest. New Drugs.

[94] Graeven U., Kremer B., Südhoff T., Killing B., Rojo F., Weber D., Tillner J., Ünal C., Schmiegel W. (2006). Phase I study of the humanised anti-EGFR monoclonal antibody matuzumab (EMD 72000) combined with gemcitabine in advanced pancreatic cancer. Br. J. Cancer.

[95] Li X., Zhao H., Gu J., Zheng L. (2016). Prognostic role of HER2 amplification based on fluorescence in situ hybridization (FISH) in pancreatic ductal adenocarcinoma (PDAC): A meta-analysis. World J. Surg. Oncol..

[96] Vu T., Claret F.X. (2012). Trastuzumab: Updated Mechanisms of Action and Resistance in Breast Cancer. Front. Oncol..

[97] Assenat E., Azria D., Mollevi C., Guimbaud R., Tubiana-Mathieu N., Smith D., Delord J.P., Samalin E., Portales F., Larbouret C. (2015). Dual targeting of HER1/EGFR and HER2 with cetuximab and trastuzumab in patients with metastatic pancreatic cancer after gemcitabine failure: Results of the “THERAPY”phase 1-2 trial. Oncotarget.

[98] Harder J., Ihorst G., Heinemann V., Hofheinz R., Moehler M., Buechler P., Kloeppel G., Röcken C., Bitzer M., Boeck S. (2012). Multicentre phase II trial of trastuzumab and capecitabine in patients with HER2 overexpressing metastatic pancreatic cancer. Br. J. Cancer.

[99] Ferrara N., Adamis A.P. (2016). Ten years of anti-vascular endothelial growth factor therapy. Nat. Rev. Drug. Discov..

[100] Van Cutsem E., Vervenne W.L., Bennouna J., Humblet Y., Gill S., Van Laethem J.L., Verslype C., Scheithauer W., Shang A., Cosaert J. (2009). Phase III trial of bevacizumab in combination with gemcitabine and erlotinib in patients with metastatic pancreatic cancer. J. Clin. Oncol..

[101] Kindler H.L., Niedzwiecki D., Hollis D., Sutherland S., Schrag D., Hurwitz H., Innocenti F., Mulcahy M.F., O’Reilly E., Wozniak T.F. (2010). Gemcitabine plus bevacizumab compared with gemcitabine plus placebo in patients with advanced pancreatic cancer: Phase III trial of the Cancer and Leukemia Group B (CALGB 80303). J. Clin. Oncol..

[102] Johansson H., Andersson R., Bauden M., Hammes S., Holdenrieder S., Ansari D. (2016). Immune checkpoint therapy for pancreatic cancer. World J. Gastroenterol..

[103] Bengsch F., Knoblock D.M., Liu A., McAllister F., Beatty G.L. (2017). CTLA-4/CD80 pathway regulates T cell infiltration into pancreatic cancer. Cancer Immunol. Immunother..

[104] Royal R.E., Levy C., Turner K., Mathur A., Hughes M., Kammula U.S., Sherry R.M., Topalian S.L., Yang J.C., Lowy I. (2010). Phase 2 Trial of Single Agent Ipilimumab (Anti-CTLA-4) for Locally Advanced or Metastatic Pancreatic Adenocarcinoma. J. Immunother..

[105] Le D.T., Lutz E., Uram J.N., Sugar E.A., Onners B., Solt S., Zheng L., Diaz Jr L.A., Donehower R.C., Jaffee E.M. (2013). Evaluation of Ipilimumab in Combination with Allogeneic Pancreatic Tumor Cells Transfected With a GM-CSF Gene in Previously Treated Pancreatic Cancer. J. Immunother..

[106] Aglietta M., Barone C., Sawyer M.B., Moore M.J., Miller W.H., Bagalà C., Colombi F., Cagnazzo C., Gioeni L., Wang E. (2014). A phase I dose escalation trial of tremelimumab (CP-675,206) in combination with gemcitabine in chemotherapy-naive patients with metastatic pancreatic cancer. Ann. Oncol..

[107] Sharma P., Dirix L., De Vos F.Y.F.L., Allison J.P., Decoster L., Zaucha R., Park J.O., Vanderwalde A.M., Kataria R.S., Ferro S. (2018). Efficacy and tolerability of tremelimumab in patients with metastatic pancreatic ductal adenocarcinoma. J. Clin. Oncol..

[108] Gong J., Chehrazi-Raffle A., Reddi S., Salgia R. (2018). Development of PD-1 and PD-L1 inhibitors as a form of cancer immunotherapy: A comprehensive review of registration trials and future considerations. J. Immunother. Cancer.

[109] Mahalingam D., Wilkinson G.A., Eng K.H., Fields P., Raber P., Moseley J.L., Cheetham K., Coffey M., Nuovo G., Kalinski P. (2020). Pembrolizumab in combination with the oncolytic virus pelareorep and chemotherapy in patients with advanced pancreatic adenocarcinoma: A Phase 1b study. Clin. Cancer Res..

[110] Weiss G.J., Blaydorn L., Beck J., Bornemann-Kolatzki K., Urnovitz H., Schütz E., Khemka V. (2017). Phase Ib/II study of gemcitabine, nab-paclitaxel, and pembrolizumab in metastatic pancreatic adenocarcinoma. Invest. New Drugs.

[111] Bockorny B., Semenisty V., Macarulla T., Borazanci E., Wolpin B.M., Stemmer S.M., Golan T., Geva R., Borad M.J., Pedersen K.S. (2020). BL-8040, a CXCR4 antagonist, in combination with pembrolizumab and chemotherapy for pancreatic cancer: The COMBAT trial. Nat. Med..

[112] Wainberg Z.A., Hochster H.S., Kim E.J., George B., Kaylan A., Chiorean E.G., Waterhouse D.M., Guiterrez M., Parikh A., Jain R. (2020). Open-label, Phase I Study of Nivolumab Combined with nab-Paclitaxel Plus Gemcitabine in Advanced Pancreatic Cancer. Clin. Cancer Res..

[113] Muñoz-Unceta N., Burgueño I., Jiménez E., Paz-Ares L. (2018). Durvalumab in NSCLC: Latest evidence and clinical potential. Ther. Adv. Med. Oncol..

[114] O’Reilly E.M., Oh D.-Y., Dhani N., Renouf D.J., Lee M.A., Sun W., Fisher G., Hezel A., Chang S.C., Vlahovic G. (2019). Durvalumab With or Without Tremelimumab for Patients with Metastatic Pancreatic Ductal Adenocarcinoma: A Phase 2 Randomized Clinical Trial. JAMA Oncol..

[115] Jakubowski C., Thompson E.D., Wang H., Walker R., Jaffee E.M., Azad N.S. (2020). A phase II trial of PD-1 inhibition with INCMGA00012 in patients with previously treated unresectable or metastatic adenosquamous pancreatic cancer. J. Clin. Oncol..

[116] Srinivasa P.P., Zhihong X., David G., Romano C.P., Jeremy S.W., Minoti V.A. (2020). Targeting HGF/c-MET Axis in Pancreatic Cancer. Int. J. Mol. Sci..

[117] Xu Z., Pang T.C.Y., Liu A.C., Pothula S.P., Mekapogu A.R., Perera C.J., Murakami T., Goldstein D., Pirola R.C., Wilson J.S. (2020). Targeting the HGF/c-MET pathway in advanced pancreatic cancer: A key element of treatment that limits primary tumour growth and eliminates metastasis. Br. J. Cancer.

[118] Rizwani W., Allen A.E., Trevino J.G. (2015). Hepatocyte growth factor from a clinical perspective: A pancreatic cancer challenge. Cancers.

[119] Patnaik A., Weiss G.J., Papadopoulos K.P., Hofmeister C.C., Tibes R., Tolcher A., Isaacs R., Jac J., Han M., Payumo F.C. (2014). Phase I ficlatuzumab monotherapy or with erlotinib for refractory advanced solid tumours and multiple myeloma. Br. J. Cancer.

[120] Tanaka N., Yamada S., Sonohara F., Suenaga M., Hayashi M., Takami H., Niwa Y., Hattori N., Iwata N., Kanda M. (2018). Clinical Implications of Lysyl Oxidase-Like Protein 2 Expression in Pancreatic Cancer. Sci. Rep..

[121] Benson A.B., Wainberg Z.A., Hecht J.R., Vyushkov D., Dong H., Bendell J., Kudrik F. (2017). A Phase II Randomized, Double-Blind, Placebo-Controlled Study of Simtuzumab or Placebo in Combination with Gemcitabine for the First-Line Treatment of Pancreatic Adenocarcinoma. Oncologist.

[122] Park J.S., Lee J.-H., Lee Y.S., Kim J.K., Dong S.M., Yoon D.S. (2016). Emerging role of LOXL2 in the promotion of pancreas cancer metastasis. Oncotarget.

[123] Yuan X., Gajan A., Chu Q., Xiong H., Wu K., Wu G.S. (2018). Developing TRAIL/TRAIL death receptor-based cancer therapies. Cancer Metastasis Rev..

[124] Kaplan-Lefko P.J., Graves J.D., Zoog S.J., Pan Y., Wall J., Branstetter D.G., Moriguchi J., Coxon A., Huard J.N., Xu R. (2010). Conatumumab, a fully human agonist antibody to death receptor 5, induces apoptosis via caspase activation in multiple tumor types. Cancer Biol. Ther..

[125] Forero-Torres A., Shah J., Wood T., Posey J., Carlisle R., Copigneaux C., Luo F., Wojtowicz-Praga S., Percent I., Saleh M. (2010). Phase I Trial of Weekly Tigatuzumab, an Agonistic Humanized Monoclonal Antibody Targeting Death Receptor 5 (DR5). Cancer Biother. Radiopharm..

[126] Forero-Torres A., Infante J.R., Waterhouse D., Wong L., Vickers S., Arrowsmith E., He A.R., Hart L., Trent D., Wade J. (2013). Phase 2, multicenter, open-label study of tigatuzumab (CS-1008), a humanized monoclonal antibody targeting death receptor 5, in combination with gemcitabine in chemotherapy-naive patients with unresectable or metastatic pancreatic cancer. Cancer Med..

[127] Luo G., Jin K., Deng S., Cheng H., Fan Z., Gong Y., Qian Y., Huang Q., Ni Q., Liu C. (2020). Roles of CA19-9 in pancreatic cancer: Biomarker, predictor and promoter. Biochim. Biophys. Acta Rev. Cancer.

[128] Poruk K.E., Gay D.Z., Brown K., Mulvihill J.D., Boucher K.M., Scaife C.L., Firpo M.A., Mulvihill S.J. (2013). The clinical utility of CA 19-9 in pancreatic adenocarcinoma: Diagnostic and prognostic updates. Curr. Mol. Med..

[129] Lohrmann C., O’Reilly E.M., O’Donoghue J.A., Pandit-Taskar N., Carrasquillo J.A., Lyashchenko S.K., Ruan S., Teng R., Scholz W., Maffuid P.W. (2019). Retooling a Blood-Based Biomarker: Phase I Assessment of the High-Affinity CA19-9 Antibody HuMab-5B1 for Immuno-PET Imaging of Pancreatic Cancer. Clin. Cancer Res..

[130] Almhanna K., Kalebic T., Cruz C., Faris J.E., Ryan D.P., Jung J., Wyant T., Fasanmade A.A., Messersmith W., Rodon J. (2016). Phase I study of the investigational anti-guanylyl cyclase antibody-drug conjugate TAK-264 (MLN0264) in adult patients with advanced gastrointestinal malignancies. Clin. Cancer Res..

[131] Schreiber A.R., Nguyen A., Bagby S.M., Arcaroli J.J., Yacob B.W., Quackenbush K., Guy J.L., Crowell T., Stringer B., Danaee H. (2018). Evaluation of TAK-264, an Antibody-Drug Conjugate in Pancreatic Cancer Cell Lines and Patient-Derived Xenograft Models. Clin. Cancer Drugs.

[132] Almhanna K., Wright D., Mercade T.M., Van Laethem J.-L., Gracian A.C., Guillen-Ponce C., Faris J., Lopez C.M., Hubner R.A., Bendell J. (2017). A phase II study of antibody-drug conjugate, TAK-264 (MLN0264) in previously treated patients with advanced or metastatic pancreatic adenocarcinoma expressing guanylyl cyclase C. Invest. New Drugs.

[133] Coveler A.L., Ko A.H., Catenacci D.V.T., Von Hoff D., Becerra C., Whiting N.C., Yang J., Wolpin B. (2016). A phase 1 clinical trial of ASG-5ME, a novel drug-antibody conjugate targeting SLC44A4, in patients with advanced pancreatic and gastric cancers. Investig. New Drugs.

[134] Piechutta M., Berghoff A.S. (2019). New emerging targets in cancer immunotherapy: The role of Cluster of Differentiation 40 (CD40/TNFR5). ESMO Open.

[135] Beatty G.L., Torigian D.A., Chiorean E.G., Saboury B., Brothers A., Alavi A., Troxel A.B., Sun W., Teitelbaum U.R., Vonderheide R.H. (2013). A Phase I Study of an Agonist CD40 Monoclonal Antibody (CP-870,893) in Combination with Gemcitabine in Patients with Advanced Pancreatic Ductal Adenocarcinoma. Clin. Cancer Res..

[136] Yang X., Guo Z., Liu Y., Si T., Yu H., Li B., Tian W. (2014). Prostate stem cell antigen and cancer risk, mechanisms and therapeutic implications. Expert Rev. Anticancer Ther..

[137] Saeki N., Gu J., Yoshida T., Wu X. (2010). Prostate stem cell antigen: A Jekyll and Hyde molecule?. Clin. Cancer Res..

[138] Wolpin B.M., O’Reilly E.M., Ko Y.J., Blaszkowsky L.S., Rarick M., Rocha-Lima C.M., Ritch P., Chan E., Spratlin J., Macarulla T. (2013). Global, multicenter, randomized, phase II trial of gemcitabine and gemcitabine plus AGS-1C4D4 in patients with previously untreated, metastatic pancreatic cancer. Ann. Oncol..

[139] Pelzer U., Bendell J.C., Womack M.S., Bahary N., Macarulla T., Borazanci E.H., Levy D.E., Mo G., Ramage S.C., Garrido-Laguna I. (2019). A phase Ib study evaluating olaratumab in combination with nab-paclitaxel and gemcitabine in first-line treatment of metastatic pancreatic cancer. J. Clin. Oncol..

[140] Reilly E.M., Wang J.S.-Z., Yu K.H., Lowery M.A., Varghese A.M., Bendell J.C., Borazanci E.H., Estrella H., Fowler K., Hoskins M. (2018). Abstract LB-B25: Preliminary phase I data comparing HuMab-5B1 (MVT-5873), a monoclonal antibody targeting sLea, as a single agent and in combination with first line nab-paclitaxel and gemcitabine in patients with CA19-9 positive pancreatic cancer. Mol. Cancer Ther..

[141] Berlin J.D., Feng Y., Catalano P., Abbruzzese J.L., Philip P.A., McWilliams R.R., Lowy A.M., Benson III A.B., Blackstock A.W. (2018). An Intergroup Randomized Phase II Study of Bevacizumab or Cetuximab in Combination with Gemcitabine and in Combination with Chemoradiation in Patients with Resected Pancreatic Carcinoma: A Trial of the ECOG-ACRIN Cancer Research Group (E2204). Oncology.

[142] Beg M.S., Azad N.S., Patel S.P., Torrealba J., Mavroukakis S., Beatson M.A., Wang X.P., Arlen P.M., Morse M.A. (2016). A phase 1 dose-escalation study of NEO-102 in patients with refractory colon and pancreatic cancer. Cancer Chemother. Pharmacol..

[143] Chadha A.S., Skinner H.D., Gunther J.R., Munsell M.F., Das P., Minsky B.D., Delclos M.E., Chatterjee D., Wang H., Clemons M. (2016). Phase i trial of consolidative radiotherapy with concurrent bevacizumab, erlotinib and capecitabine for unresectable pancreatic cancer. PLoS ONE.

[144] Khan K., Cunningham D., Peckitt C., Barton S., Tait D., Hawkins M., Watkins D., Starling N., Rao S., Begum R. (2016). miR-21 expression and clinical outcome in locally advanced pancreatic cancer: Exploratory analysis of the pancreatic cancer Erbitux, radiotherapy and UFT (PERU) trial. Oncotarget.

[145] Fiore M., Trodella L., Valeri S., Borzomati D., Floreno B., Ippolito E., Trecca P., Trodella L.E., D’Angelillo R.M., Ramella S. (2015). Prospective study of cetuximab and gemcitabine in combination with radiation therapy: Feasibility and efficacy in locally advanced pancreatic head cancer. Radiat. Oncol..

[146] Picozzi V.J., Ramanathan R.K., Lowery M.A., Ocean A.J., Mitchel E.P., O’Neil B.H., Guarino M.J., Conkling P.R., Cohen S.J., Bahary N. (2015). (90)Y-clivatuzumab tetraxetan with or without low-dose gemcitabine: A phase Ib study in patients with metastatic pancreatic cancer after two or more prior therapies. Eur. J. Cancer.

[147] Okusaka T., Ikeda M., Fukutomi A., Kobayashi Y., Shibayama K., Takubo T., Gansert J. (2014). Safety, Tolerability, Pharmacokinetics and Antitumor Activity of Ganitumab, an Investigational Fully Human Monoclonal Antibody to Insulin-like Growth Factor Type 1 Receptor, Combined with Gemcitabine as First-line Therapy in Patients with Metastatic Pancreatic Cancer: A Phase 1b Study. Jpn. J. Clin. Oncol..

[148] Sahora K., Schindl M., Kuehrer I., Eisenhut A., Werba G., Brostjan C., Telek B., Ba’ssalamah A., Stift J., Schoppmann S.F. (2014). A phase II trial of two durations of Bevacizumab added to neoadjuvant gemcitabine for borderline and locally advanced pancreatic cancer. Anticancer Res..

[149] Watkins D.J., Starling N., Cunningham D., Thomas J., Webb J., Brown G., Barbachano Y., Oates J., Chau I. (2014). The combination of a chemotherapy doublet (gemcitabine and capecitabine) with a biological doublet (bevacizumab and erlotinib) in patients with advanced pancreatic adenocarcinoma. The results of a phase I/II study. Eur. J. Cancer.

[150] Su D., Jiao S.-C., Wang L.-J., Shi W.-W., Long Y.-Y., Li J., Bai L. (2014). Efficacy of nimotuzumab plus gemcitabine usage as first-line treatment in patients with advanced pancreatic cancer. Tumour Biol..

[151] Van Buren Ii G., Ramanathan R.K., Krasinskas A.M., Smith R.P., Abood G.J., Bahary N., Lembersky B.C., Shuai Y., Potter D.M., Bartlett D.L. (2013). Phase II Study of Induction Fixed-Dose Rate Gemcitabine and Bevacizumab Followed by 30 Gy Radiotherapy as Preoperative Treatment for Potentially Resectable Pancreatic Adenocarcinoma. Ann. Surg. Oncol..

[152] Kordes S., Richel D.J., Klümpen H.-J., Weterman M.J., Stevens A.J.W.M., Wilmink J.W. (2013). A phase I/II, non-randomized, feasibility/safety and efficacy study of the combination of everolimus, cetuximab and capecitabine in patients with advanced pancreatic cancer. Invest. New Drugs.

[153] Martin L.K., Li X., Kleiber B., Ellison E.C., Bloomston M., Zalupski M., Bekaii-Saab T.S. (2012). VEGF remains an interesting target in advanced pancreas cancer (APCA): Results of a multi-institutional phase II study of bevacizumab, gemcitabine, and infusional 5-fluorouracil in patients with APCA. Ann. Oncol..

[154] Pipas J.M., Zaki B.I., McGowan M.M., Tsapakos M.J., Ripple G.H., Suriawinata A.A., Tsongalis G.J., Colacchio T.A., Gordon S.R., Sutton J.E. (2012). Neoadjuvant cetuximab, twice-weekly gemcitabine, and intensity-modulated radiotherapy (IMRT) in patients with pancreatic adenocarcinoma. Ann. Oncol..

[155] Ocean A.J., Pennington K.L., Guarino M.J., Sheikh A., Bekaii-Saab T., Serafini A.N. (2012). Fractionated radioimmunotherapy with (90) Y-clivatuzumab tetraxetan and low-dose gemcitabine is active in advanced pancreatic cancer: A phase 1 trial. Cancer.

[156] Ko A.H., Youssoufian H., Gurtler J., Dicke K., Kayaleh O., Lenz H.-J., Keaton M., Katz T., Ballal S., Rowinsky E.K. (2012). A phase II randomized study of cetuximab and bevacizumab alone or in combination with gemcitabine as first-line therapy for metastatic pancreatic adenocarcinoma. Invest. New Drugs.

[157] Merchan J.R., Ferrell A., Macintyre J., Ciombor K.K., Levi J., Ribeiro A., Sleeman D., Flores A., Lopes G., Rocha-Lima C.M. (2012). Phase II study of gemcitabine, oxaliplatin, and cetuximab in advanced pancreatic cancer. Am. J. Clin. Oncol..

[158] Kullmann F., Hartmann A., Stöhr R., Messmann H., Dollinger M.M., Trojan J., Fuchs M., Hollerbach S., Harder J., Troppmann M. (2011). KRAS mutation in metastatic pancreatic ductal adenocarcinoma: Results of a multicenter phase II study evaluating efficacy of cetuximab plus gemcitabine/oxaliplatin (GEMOXCET) in first-line therapy. Oncology.

[159] Crane C.H., Varadhachary G.R., Yordy J.S., Staerkel G.A., Javle M.M., Safran H., Haque W., Hobbs B.D., Krishnan S., Fleming J.B. (2011). Phase II trial of cetuximab, gemcitabine, and oxaliplatin followed by chemoradiation with cetuximab for locally advanced (T4) pancreatic adenocarcinoma: Correlation of Smad4(Dpc4) immunostaining with pattern of disease progression. J. Clin. Oncol..

[160] Gulec S.A., Cohen S.J., Pennington K.L., Zuckier L.S., Hauke R.J., Horne H., Wegener W.A., Teoh N., Gold D.V., Sharkey R.M. (2011). Treatment of advanced pancreatic carcinoma with 90Y-clivatuzumab tetraxetan: A phase I single-dose escalation trial. Clin. Cancer Res..

[161] Fogelman D., Jafari M., Varadhachary G.R., Xiong H., Bullock S., Ozer H., Lin E., Morris J., Cunningham P., Bennett B. (2011). Bevacizumab plus gemcitabine and oxaliplatin as first-line therapy for metastatic or locally advanced pancreatic cancer: A phase II trial. Cancer Chemother. Pharmacol..

[162] Small W., Mulcahy M.F., Rademaker A., Bentrem D.J., Benson A.B., Weitner B.B., Talamonti M.S. (2011). Phase II trial of full-dose gemcitabine and bevacizumab in combination with attenuated three-dimensional conformal radiotherapy in patients with localized pancreatic cancer. Int. J. Radiat. Oncol. Biol. Phys..

[163] Arnoletti J.P., Frolov A., Eloubeidi M., Keene K., Posey J., Wood T., Greeno E., Jhala N., Varadarajulu S., Russo S. (2011). A phase I study evaluating the role of the anti-epidermal growth factor receptor (EGFR) antibody cetuximab as a radiosensitizer with chemoradiation for locally advanced pancreatic cancer. Cancer Chemother. Pharmacol..

[164] Astsaturov I., Meropol N., Alpaugh R., Burtness B., Cheng J., McLaughlin S., Rogatko A., Xu Z., Watson J.C., Weiner L.M. (2011). Phase II and coagulation cascade biomarker study of bevacizumab with or without docetaxel in patients with previously treated metastatic pancreatic adenocarcinoma. Am. J. Clin. Oncol..

[165] Ko A.H., Venook A.P., Bergsland E.K., Kelley R.K., Korn W.M., Dito E., Schillinger B., Scott J., Hwang J., Tempero M.A. (2010). A phase II study of bevacizumab plus erlotinib for gemcitabine-refractory metastatic pancreatic cancer. Cancer Chemother. Pharmacol..

[166] Starling N., Watkins D., Cunningham D., Thomas J., Webb J., Brown G., Thomas K., Oates J., Chau I. (2009). Dose finding and early efficacy study of gemcitabine plus capecitabine in combination with bevacizumab plus erlotinib in advanced pancreatic cancer. J. Clin. Oncol..

[167] Crane C.H., Winter K., Regine W.F., Safran H., Rich T.A., Curran W., Wolff R.A., Willett C.G. (2009). Phase II study of bevacizumab with concurrent capecitabine and radiation followed by maintenance gemcitabine and bevacizumab for locally advanced pancreatic cancer: Radiation Therapy Oncology Group RTOG 0411. J. Clin. Oncol..

[168] Javle M., Yu J., Garrett C., Pande A., Kuvshinoff B., Litwin A., Phelan J., Gibbs J., Iyer R. (2009). Bevacizumab combined with gemcitabine and capecitabine for advanced pancreatic cancer: A phase II study. Br. J. Cancer.

[169] Kullmann F., Hollerbach S., Dollinger M.M., Harder J., Fuchs M., Messmann H., Trojan J., Gäbele E., Hinke A., Hollerbach C. (2009). Cetuximab plus gemcitabine oxaliplatin (GEMOXCET) in first-line metastatic pancreatic cancer: A multicentre phase II study. Br. J. Cancer.

[170] Sultana A., Shore S., Raraty M.G., Vinjamuri S., Evans J.E., Smith C.T., Lane S., Chauhan S., Bosonnet L., Garvey C. (2009). Randomised Phase I/II trial assessing the safety and efficacy of radiolabelled anti-carcinoembryonic antigen I(131) KAb201 antibodies given intra-arterially or intravenously in patients with unresectable pancreatic adenocarcinoma. BMC Cancer.

[171] Ko A.H., Dito E., Schillinger B., Venook A.P., Xu Z., Bergsland E.K., Wong D., Scott J., Hwang J., Tempero M.A. (2008). A phase II study evaluating bevacizumab in combination with fixed-dose rate gemcitabine and low-dose cisplatin for metastatic pancreatic cancer: Is an anti-VEGF strategy still applicable?. Invest. New Drugs.

[172] Cascinu S., Berardi R., Labianca R., Siena S., Falcone A., Aitini E., Barni S., Di Costanzo F., Dapretto E., Tonini G. (2008). Cetuximab plus gemcitabine and cisplatin compared with gemcitabine and cisplatin alone in patients with advanced pancreatic cancer: A randomised, multicentre, phase II trial. Lancet Oncol..

[173] Crane C.H., Ellis L.M., Abbruzzese J.L., Amos C., Xiong H.Q., Ho L., Evans D.B., Tamm E.P., Ng C., Pisters P.W. (2006). Phase I trial evaluating the safety of bevacizumab with concurrent radiotherapy and capecitabine in locally advanced pancreatic cancer. J. Clin. Oncol..

[174] Kindler H.L., Friberg G., Singh D.A., Locker G., Nattam S., Kozloff M., Taber D.A., Karrison T., Dachman A., Stadler W.M. (2005). Phase II Trial of Bevacizumab Plus Gemcitabine in Patients with Advanced Pancreatic Cancer. J. Clin. Oncol..

[175] Xiong H.Q., Rosenberg A., LoBuglio A., Schmidt W., Wolff R.A., Deutsch J., Needle M., Abbruzzese J.L. (2004). Cetuximab, a Monoclonal Antibody Targeting the Epidermal Growth Factor Receptor, in Combination With Gemcitabine for Advanced Pancreatic Cancer: A Multicenter Phase II Trial. J. Clin. Oncol..

[176] Weiner L.M., Harvey E., Padavic-Shaller K., Willson J.K.V., Walsh C., LaCreta F., Khazaeli M.B., Kirkwood J.M., Haller D.G. (1993). Phase II Multicenter Evaluation of Prolonged Murine Monoclonal Antibody 17-1A Therapy in Pancreatic Carcinoma. J. Immunother..

[177] Zhang Y., Yang C., Cheng H., Fan Z., Huang Q., Lu Y., Fan K., Luo G., Jin K., Wang Z. (2018). Novel agents for pancreatic ductal adenocarcinoma: Emerging therapeutics and future directions. J. Hematol. Oncol..

[178] Ho W.J., Jaffee E.M., Zheng L. (2020). The tumour microenvironment in pancreatic cancer—clinical challenges and opportunities. Nat. Rev. Clin. Oncol..

[179] Hosein A.N., Brekken R.A., Maitra A. (2020). Pancreatic cancer stroma: An update on therapeutic targeting strategies. Nat. Rev. Gastroenterol. Hepatol..

[180] Roth M.T., Cardin D.B., Berlin J.D. (2020). Recent advances in the treatment of pancreatic cancer. F1000Research.

[181] Khan T., Seddon A.M., Dalgleish A.G., Khelwatty S., Ioannou N., Mudan S., Modjtahedi H. (2020). Synergistic activity of agents targeting growth factor receptors, CDKs and downstream signaling molecules in a panel of pancreatic cancer cell lines and the identification of antagonistic combinations: Implications for future clinical trials in pancreatic cancer. Oncol. Rep..

[182] Wolchok J.D., Chiarion-Sileni V., Gonzalez R., Rutkowski P., Grob J.-J., Cowey C.L., Lao C.D., Wagstaff J., Schadendorf D., Ferrucci P.F. (2017). Overall Survival with Combined Nivolumab and Ipilimumab in Advanced Melanoma. N. Engl. J. Med..

[183] Hodi F.S., O’Day S.J., McDermott D.F., Weber R.W., Sosman J.A., Haanen J.B., Gonzalez R., Robert C., Schadendorf D., Hassel J.C. (2010). Improved Survival with Ipilimumab in Patients with Metastatic Melanoma. N. Engl. J. Med..

[184] Rini B.I., Plimack E.R., Stus V., Gafanov R., Hawkins R., Nosov D., Pouliot F., Alekseev B., Soulières D., Melichar B. (2019). Pembrolizumab plus Axitinib versus Sunitinib for Advanced Renal-Cell Carcinoma. N. Engl. J. Med..

[185] Motzer R.J., Tannir N.M., McDermott D.F., Frontera O.A., Melichar B., Choueiri T.K., Plimack E.R., Barthélémy P., Porta C., George S. (2018). Nivolumab plus Ipilimumab versus Sunitinib in Advanced Renal-Cell Carcinoma. N. Engl. J. Med..

[186] Motzer R.J., Penkov K., Haanen J., Rini B., Albiges L., Campbell M.T., Venugopal B., Kollmannsberger C., Negrier S., Uemura M. (2019). Avelumab plus Axitinib versus Sunitinib for Advanced Renal-Cell Carcinoma. N. Engl. J. Med..

[187] Akce M., Zaidi M.Y., Waller E.K., El-Rayes B.F., Lesinski G.B. (2018). The Potential of CAR T Cell Therapy in Pancreatic Cancer. Front. Immunol..

[188] Neesse A., Algül H., Tuveson A.D., Gress T.M. (2015). Stromal biology and therapy in pancreatic cancer: A changing paradigm. Gut.

[189] Jiang B., Zhou L., Lu J., Wang Y., Liu C., You L., Guo J. (2020). Stroma-Targeting Therapy in Pancreatic Cancer: One Coin with Two Sides?. Front. Oncol..

[190] Li T.-J., Wang W.-Q., Yu X.-J., Liu L. (2020). Killing the “BAD”: Challenges for immunotherapy in pancreatic cancer. Biochim. Biophys. Acta Rev. Cancer..

[191] Sleightholm R.L., Neilsen B.K., Li J., Steele M.M., Singh R.K., Hollingsworth M.A., Oupicky D. (2017). Emerging roles of the CXCL12/CXCR4 axis in pancreatic cancer progression and therapy. Pharmacol. Ther..

[192] Leonardi A.J., Kotlyar D.S., Proenca R.B. CAR T cell therapy. Cancers.

[193] Maude S.L., Laetsch T.W., Buechner J., Rives S., Boyer M., Bittencourt H., Bader P., Verneris M.R., Stefanski H.E., Myers G.D. (2018). Tisagenlecleucel in Children and Young Adults with B-Cell Lymphoblastic Leukemia. N. Engl. J. Med..

[194] Neelapu S.S., Locke F.L., Bartlett N.L., Lekakis L.J., Miklos D.B., Jacobson C.A., Braunschweig I., Oluwole O.O., Siddiqi T., Lin Y. (2017). Axicabtagene Ciloleucel CAR T-Cell Therapy in Refractory Large B-Cell Lymphoma. N. Engl. J. Med..

[195] Schuster S.J., Bishop M.R., Tam C.S., Waller E.K., Borchmann P., McGuirk J.P., Jäger U., Jaglowski S., Andreadis C., Westin J.R. (2019). Tisagenlecleucel in Adult Relapsed or Refractory Diffuse Large B-Cell Lymphoma. N. Engl. J. Med..

[196] Abramson J.S., Palomba M.L., Gordon L.I., Lunning M.A., Wang M., Arnason J., Mehta A., Purev E., Maloney D.G., Andreadis C. (2020). Lisocabtagene maraleucel for patients with relapsed or refractory large B-cell lymphomas (TRANSCEND NHL 001): A multicentre seamless design study. Lancet.

[197] Jacobson C.A., Chavez J.C., Sehgal A.R., William B.M., Munoz J., Salles G.A., Casulo C., Munshi P.N., Maloney D.G., De Vos S. (2020). Interim analysis of ZUMA-5: A phase II study of axicabtagene ciloleucel (axi-cel) in patients (pts) with relapsed/refractory indolent non-Hodgkin lymphoma (R/R iNHL). J. Clin. Oncol..

[198] Munshi N.C., Anderson L.D., Shah N., Madduri D., Berdeja J., Lonial S., Raje N., Lin Y., Siegel D., Oriol A. (2021). Idecabtagene Vicleucel in Relapsed and Refractory Multiple Myeloma. N. Engl. J. Med..

[199] Haas A.R., Tanyi J.L., O’Hara M.H., Gladney W.L., Lacey S.F., Torigian D.A., Soulen M.C., Tian L., McGarvey M., Nelson A.M. (2019). Phase I Study of Lentiviral-Transduced Chimeric Antigen Receptor-Modified T Cells Recognizing Mesothelin in Advanced Solid Cancers. Mol. Ther..

[200] Li N., Li D., Ren H., Torres M., Ho M. (2019). Chimeric antigen receptor T-cell therapy targeting glypican-1 in pancreatic cancer. Cancer Res..

[201] Ma H.S., Poudel B., Torres E.R., Sidhom J.-W., Robinson T.M., Christmas B., Scott B., Cruz K., Woolman S., Wall V.Z. (2019). A CD40 Agonist and PD-1 Antagonist Antibody Reprogram the Microenvironment of Nonimmunogenic Tumors to Allow T-cell–Mediated Anticancer Activity. Cancer Immunol. Res..

[202] Liu X., Zhang M., Go V.L.W., Hu S. (2010). Membrane proteomic analysis of pancreatic cancer cells. J. Biomed. Sci..

[203] Hu Z.I., Bendell J.C., Bullock A., LoConte N.K., Hatoum H., Ritch P., Hool H., Leach J.W., Sanchez J., Sohal D.P.S. (2019). A randomized phase II trial of nab-paclitaxel and gemcitabine with tarextumab or placebo in patients with untreated metastatic pancreatic cancer. Cancer Med..

[204] Liu J.F., Moore K.N., Birrer M.J., Berlin S., Matulonis U.A., Infante J.R., Wolpin B., Poon K.A., Firestein R., Xu J. (2016). Phase I study of safety and pharmacokinetics of the anti-MUC16 antibody–drug conjugate DMUC5754A in patients with platinum-resistant ovarian cancer or unresectable pancreatic cancer. Ann. Oncol..

[205] Hoogstins C.E.S., Boogerd L.S.F., Bsc B.G.S.M., Mieog J.S.D., Swijnenburg R.J., Van De Velde C.J.H., Sarasqueta A.F., Bonsing B.A., Framery B., Pèlegrin A. (2018). Image-Guided Surgery in Patients with Pancreatic Cancer: First Results of a Clinical Trial Using SGM-101, a Novel Carcinoembryonic Antigen-Targeting, Near-Infrared Fluorescent Agent. Ann. Surg. Oncol..

[206] King J., Bouvet M., Singh G., Williams J. (2017). Improving theranostics in pancreatic cancer. J. Surg. Oncol..

[207] Dammes N., Peer D. (2020). Monoclonal antibody-based molecular imaging strategies and theranostic opportunities. Theranostics.

[208] Knutson S., Raja E., Bomgarden R., Nlend M., Chen A., Kalyanasundaram R., Desai S. (2016). Development and Evaluation of a Fluorescent Antibody-Drug Conjugate for Molecular Imaging and Targeted Therapy of Pancreatic Cancer. PLoS ONE.

[209] Moroz A., Wang Y.-H., Sharib J.M., Wei J., Zhao N., Huang Y., Chen Z., Martinko A.J., Zhuo J., Lim S.A. (2020). Theranostic Targeting of CUB Domain Containing Protein 1 (CDCP1) in Pancreatic Cancer. Clin. Cancer Res..

[210] Escorcia F.E., Houghton J.L., Abdel-Atti D., Pereira P.R., Cho A., Gutsche N.T., Baidoo K.E., Lewis J.S. (2020). ImmunoPET Predicts Response to Met-targeted Radioligand Therapy in Models of Pancreatic Cancer Resistant to Met Kinase Inhibitors. Theranostics.

[211] Sutcliffe J.L. (2020). Abstract IA-13: Molecularly targeted imaging and treatment via the integrin αvβ6. Cancer Res..

[212] Watabe T., Liu Y., Kaneda-Nakashima K., Shirakami Y., Lindner T., Ooe K., Toyoshima A., Nagata K., Shimosegawa E., Haberkorn U. (2019). Theranostics Targeting Fibroblast Activation Protein in the Tumor Stroma: 64Cu- and 225Ac-Labeled FAPI-04 in Pancreatic Cancer Xenograft Mouse Models. J. Nucl. Med..

[213] Lindner T., Altmann A., Kraemer S., Kleist C., Loktev A., Kratochwil C., Giesel F., Mier W., Marme F., Debus J. (2020). Design and Development of 99mTc-Labeled FAPI Tracers for SPECT Imaging and 188Re Therapy. J. Nucl. Med..

[214] Qiu W., Zhang H., Chen X., Song L., Cui W., Ren S., Wang Y., Guo K., Li D., Chen R. (2019). A GPC1-targeted and gemcitabine-loaded biocompatible nanoplatform for pancreatic cancer multimodal imaging and therapy. Nanomedicine.

[215] Zhou H., Qian W., Uckun F.M., Wang L., Wang Y.A., Chen H., Kooby D., Yu Q., Lipowska M., Staley C.A. (2015). IGF1 Receptor Targeted Theranostic Nanoparticles for Targeted and Image-Guided Therapy of Pancreatic Cancer. ACS Nano.

[216] Cruz E., Kayser V. (2019). Monoclonal antibody therapy of solid tumors: Clinical limitations and novel strategies to enhance treatment efficacy. Biologics.

[217] Debie P., Devoogdt N., Hernot S. (2019). Targeted Nanobody-Based Molecular Tracers for Nuclear Imaging and Image-Guided Surgery. Antibodies.

[218] Zhao X., Ning Q., Mo Z., Tang S. (2019). A promising cancer diagnosis and treatment strategy: Targeted cancer therapy and imaging based on antibody fragment. Artif. Cells Nanomed. Biotechnol..

